# Towards Fire-Safe Polymer Electrolytes for Lithium-Ion Batteries: Strategies for Electrolyte Design and Structural Design

**DOI:** 10.3390/polym17212828

**Published:** 2025-10-23

**Authors:** Khang Le Truong, Joonho Bae

**Affiliations:** Department of Physics and Semiconductor Science, Gachon University, Seongnam-si 13120, Republic of Korea; khangle123@gachon.ac.kr

**Keywords:** flame retardant, polymer electrolytes, lithium-ion batteries

## Abstract

Lithium-ion batteries, widely used in phones and electric vehicles, pose safety concerns due to the flammability of conventional liquid electrolytes, which are prone to ignition under elevated temperatures and mechanical stress, increasing the risk of fire. Polymer electrolytes have been employed as a safer solution thanks to their superior thermal stability and mechanical strength. However, despite these advantages, many polymer matrices pose a fire hazard, limiting their potential. This review assesses recent advances in enhancing the flame retardancy of polymer electrolytes through a variety of strategies, namely the incorporation of flame-retardant additives, the addition of nanoscale fillers to improve thermal resistance, and the design of layered or hybrid polymer membrane structures that function as thermal barriers. This review evaluates the effectiveness of these methods, examining their flame-retardancy as well as their influences on ionic conductivity and overall battery performance. By highlighting recent progress and enduring safety challenges in solid-state batteries, it aims to offer insights for developing lithium batteries with enhanced safety and high performance.

## 1. Introduction

Electrochemical energy storage spans multiple platforms optimized for different figures of merit. At one end are electrochemical capacitors and hybrids that emphasize power, cycle life, and intrinsic safety; at the other are high-energy rechargeable batteries for portable devices, transportation, and grids, alongside platform-specific options such as sodium-ion, lithium-metal solid-state, lithium–sulfur, metal–air, and redox-flow systems. Across this landscape, performance depends on how materials, interfaces, and architectures negotiate trade-offs among energy and power density, durability, safety, manufacturability, and cost. Polymer electrolytes fit naturally into this picture as a cross-cutting lever that can harden cells against leakage and spray ignition, enable solid-state configurations, and provide condensed-phase protection without committing to a single chemistry [1,2,3,4,5,6]. Within this ecosystem, lithium-ion batteries are fundamental to contemporary electrification, from smartphones and laptops to grid storage and electric vehicles. In 2024, global EV sales exceeded 17 million and surpassed a 20% market share, underscoring lithium-ion batteries’ central role in decarbonization and mobility [7]. Fundamental viewpoints explain the appeal and the risk. Organic electrolytes offer wide electrochemical windows and high cell voltages, while intercalation chemistries balance specific energy and cycle life. Those choices, however, couple performance to flammability hazards inherited from volatile, combustible liquid electrolytes [8,9]. The safety problem is mechanistic: conventional carbonate-based electrolytes (e.g., EC/DEC/DMC with LiPF_6_) are volatile and readily ignitable; under abuse or defect, exothermic reactions accelerate, vent flammable gases, and feed a self-heating loop known as thermal runaway [10]. Multiscale analyses show how electrical, thermal, or mechanical triggers converge on internal shorts that precipitate runaway, while fire testing quantifies heat-release rates and toxic effluents from burning cells and modules [10,11]. Even at the solvent level, measured flammability limits for common carbonates indicate narrow safety margins in air, helping explain why a single failed pouch or cylindrical cell can escalate to pack-level events [12]. Real-world incidents reinforce the point: the U.S. Department of Transportation and FAA banned Samsung Galaxy Note7 devices from air transport in October 2016 after battery fires [13], and micromobility incidents have surged in cities; for example, New York City’s FDNY recorded 277 lithium-ion battery-related fires in 2024 and expanded certification and public-safety campaigns in response [14]. On the research side, standardized failure datasets now compare heat output, ejecta, and propagation across cell formats and chemistries, strengthening the evidence base for prevention and mitigation [15]. Polymer electrolytes are pursued as a direct response to these hazards. By immobilizing or replacing free liquid within a solid or gel polymer matrix, leakage paths are eliminated, wicking is reduced, and mechanically compliant interfaces become feasible. Solid polymer electrolytes (SPEs) and gel or quasi-solid polymer electrolytes (GPEs) promise manufacturing advantages, including thinner separators, simplified packaging, and shape-conformable designs, and, in principle, improved abuse tolerance because fuel availability and spray fire modes are curtailed. The design space is rich, including host polymers such as poly(ethylene oxide) (PEO), poly (vinylidene fluoride-co-hexafluoropropylene) (PVDF-HFP), polyacrylonitrile (PAN), and polycarbonates; lithium salts including LiTFSI and LiFSI; and additives or fillers that decouple ion transport from mechanical and thermal behavior [16,17,18]. However, polymer electrolytes are not automatically nonflammable. Most organic hosts are combustible, and many GPEs retain substantial fractions of volatile solvent or plasticizer. Even nominally “solid” films can shrink, drip, or support flame spread when heated. Moreover, achieving room-temperature ionic conductivity typically pushes formulations toward softer, solvent-rich or plasticized networks that trade mechanical robustness for transport. As a result, the literature converges on a pragmatic view: polymer electrolytes mitigate leakage and spray-ignition risks and can slow propagation, but intrinsic flame resistance must be engineered into the material. Recent reviews categorize effective strategies into condensed-phase approaches (endothermic fillers, char formers, barrier-building chemistries), gas-phase mechanisms (radical scavengers), and hybrid structural designs that preserve voltage stability and rate capability [16,19,20]. This review takes that challenge as its focus. We survey flame-safety strategies specifically for polymer electrolytes in lithium batteries, spanning solvent-free SPEs, quasi-solid and gel systems, and composite polymer electrolytes that incorporate inorganic and molecular additives, while treating performance holistically. Alongside flammability metrics (limiting oxygen index, thermogravimetric analysis, UL-94 ratings, cone calorimetry, self-extinguishing time), we evaluate thermal-runaway suppression and propagation at the cell and module scales, and the electrochemical figures of merit any safe electrolyte must deliver: room-temperature ionic conductivity, Li^+^ transference number, interfacial stability with Li metal and high-voltage cathodes, and durable cycling under realistic test protocols. By connecting safety science from abuse testing and incident analyses to polymer chemistry and interfacial physics, we aim to map materials choices that turn safety from an incidental outcome into a property architected from the molecular level to the battery pack [10,16,19,20].

## 2. Thermal Runaway in Lithium-Ion Cells: Mechanisms and Implications for Polymer Electrolytes

**Figure 1:**
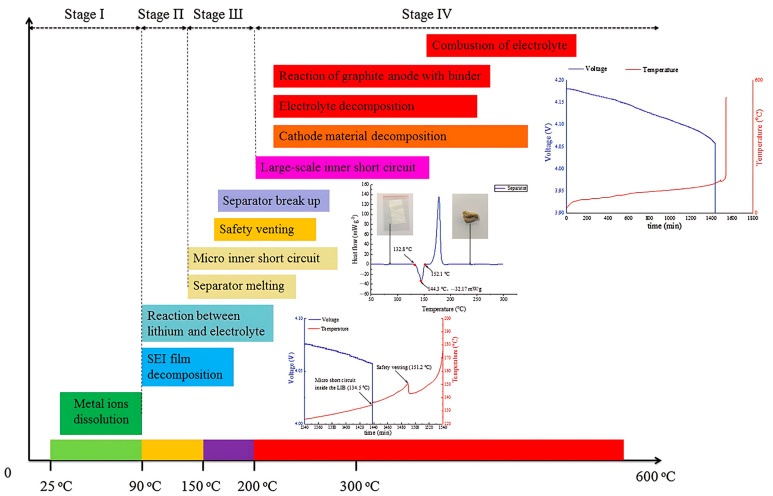
Staged progression of thermal runaway under heating. Reproduced with permission from Ref. [35] (Elsevier, 2019).

Thermal runaway (TR) in lithium-ion cells is a self-accelerating cascade of exothermic reactions initiated by abuse conditions such as overheating, overcharge, crushing, or internal shorting. Once a critical temperature is crossed, heat generation exceeds dissipation, leading to rapid temperature rise, venting of flammable gases, ignition, and, under confinement, possible explosion. In conventional cells, organic carbonate electrolytes serve as both fuel and reaction medium, while a charged cathode supplies oxidizing equivalents, so the event can sustain itself [10,21,22]. The onset is often described in four overlapping phases. Phase I (≈60–120 °C): destabilization of the anode Solid Electrolyte Interphase (SEI) exposes lithiated carbon to fresh electrolyte, parasitic reductions evolve H_2_, C_2_H_4_, and light hydrocarbons, and LiPF_6_ decomposition to PF_5_/POF_3_ with HF erodes the SEI/Cathode Electrolyte Interphase (CEI) and accelerates side reactions [23,24,25]. Phase II (≈120–150 °C): polyolefin separators shrink or melt (polyethylene (PE) ≈ 130 °C, Polypropylene (PP) ≈ 160 °C), temporary pore shutdown yields to mechanical failure and soft shorts that intensify local heating [10,26,27]. Phase III (≈130–200 °C): continued anode–electrolyte exotherms coincide with oxygen-releasing breakdown of layered-oxide cathodes, sharply increasing heat-release rate and internal pressure as organic vapors are oxidized [28,29,30]. Phase IV (>200 °C): lattice collapse and O_2_ release couple with rich fuel vapors to produce flame jets and pressure pulses, with pack-level hazard governed by gas yield and confinement [21,28,31]. This four-phase sequence is summarized in Figure 1.

SEI/electrolyte chemistry, separator mechanics, and cathode oxygen release form a tight feedback loop, with the liquid electrolyte supplying the dominant volatile fuel and thus setting failure severity. Polymer electrolytes mitigate this by replacing carbonates with a largely non-volatile matrix that removes most gas-phase fuel, localizes failure, and enables condensed-phase protection (char, intumescence, ceramization) when phosphorus or nitrogen motifs and inorganic fillers are used; higher modulus and intimate contact in in situ or composite SPEs further stabilize interfaces and microstructure [16,17,19,32]. But “solid” is not “fire-proof”: many backbones are combustible and can pyrolyze to fragments readily oxidized by cathode-released oxygen, and TR has been documented in all-solid cells, including millisecond events with overpressure, hot gases, flames, and incandescent ejecta [19,33]. Design must balance three linked trade-offs: ion transport (dry SPEs show low room-temperature conductivity and modest tLi^+^; plasticizers or ionic liquids raise transport but reintroduce volatility or combustibility) [32,34], interfaces (forming low-resistance, stable interphases with Li metal and high-voltage cathodes is difficult and can evolve heat or gas) [34], and oxidizer coupling (Ni-rich layered oxides release lattice oxygen at high temperature and high state of charge, accelerating polymer oxidation and narrowing safety margins even without liquid solvent) [28,29,30]. In sum, SPEs offer intrinsic safety advantages over liquids, but only purpose-built, flame-resistant architectures translate materials-level gains into cell- and pack-level safety.

## 3. Safety Assessment of Polymer Electrolytes

### 3.1. Flammability Tests

Flammability testing first establishes a controlled materials-level baseline for SPEs, isolating intrinsic ignitability, heat-release behavior, gas evolution, and condensed-phase protection under standardized conditions. The next subsections outline four complementary methods that span rapid ignitability/self-extinction screening to quantitative calorimetry and thermoanalysis, and concisely list their key readouts and reporting practices.

#### 3.1.1. Self-Extinguishing Time (SET)

Self-Extinguishing Time (SET) quantifies self-extinguishing as after-flame time normalized by combustible mass (SET = t/m, s·g^−1^). It provides a rapid, film-level screen for thin free-standing or gel-impregnated membranes and is widely used in the electrolyte literature following the practice introduced by Xu and co-workers. In screening at comparable areal mass, <6 s·g^−1^ is conventionally classified as material-level nonflammable, 6–20 s·g^−1^ as flame-retarded, and >20 s·g^−1^ as flammable. The <6 s·g^−1^ boundary is empirical for thin films; values in this range often co-occur with UL-94 vertical ratings (VTM-0 or V-0) at similar thickness, but the two tests are not interchangeable. UL-94 vertical (V for self-supporting films; VTM for very thin films) categorizes after-flame, after-glow, and flaming drips that can ignite cotton. Because SET can drift with areal mass, imbibed volatiles, fixture heat-sinking, and edge geometry, results should include thickness/areal mass, imbibed loading, mounting/backing, specimen geometry, replicate counts, and uncertainty. In practice, SET and UL-94 serve as fast screens that are corroborated with cone calorimetry and cell-level abuse tests [34,36,37,38,39].

#### 3.1.2. Limiting Oxygen Index

Limiting Oxygen Index (LOI) is measured according to ASTM D2863 [40] or ISO 4589 (series); Plastics–Determination of burning behaviour by oxygen index (ISO 4589-2: Ambient-temperature test; ISO 4589-3: Elevated-temperature test). International Organization for Standardization: Geneva, Switzerland, 2017. It is defined as the minimum volume fraction of oxygen in an O_2_/N_2_ stream that just sustains flaming of a vertical strip. LOI provides a quick benchmark against air at about 20.95% O_2_ and is highly sensitive to formulation, which is why it is widely used for screening. Interpreted at room conditions in the polymer literature, values at or below 20.9% indicate flammability in air, values around 21–27% indicate slow-burning or combustible behavior in the screening sense, and values at or above roughly 28% are commonly treated as self-extinguishing by convention. LOI is a material-level screen rather than a predictor of end-use fire behavior, so it is typically considered alongside cone calorimetry and, in battery research, cell-level abuse evaluations [41,42,43].

#### 3.1.3. Cone Calorimetry

Under fixed radiant flux (typically 25–50 kW m^−2^), cone calorimetry applies oxygen-consumption calorimetry to deliver heat-release rate (HRR) curves, peak HRR (pHRR), total heat release (THR), time-to-ignition (TTI), mass loss, and smoke, the bench-scale reference for assessing condensed-phase protection in SPE laminates. For thin membranes, reporting flux, orientation, mounting/backing/edge sealing, and thickness/areal density is essential, so pHRR/THR reflect intrinsic behavior rather than fixture artifacts [44,45].

#### 3.1.4. Thermogravimetric Analysis

TGA separates volatility from backbone decomposition by tracking mass loss under controlled atmospheres, reporting T_onset_ (e.g., 5% loss), DTG peak (T_max_), and final residue (char/ash). For salt/filler-rich SPEs, an ash-corrected residue should be provided so inorganic ash is not misread as protective carbon; when needed, sequential inert and oxidative runs should be used to quantify ash. At minimum, one should report atmosphere and flow, heating rate/program, sample mass and pan/crucible, terminal hold for residue, and replicate statistics [46,47,48].

In summary, LOI, SET, cone calorimetry, and TGA provide a tiered framework for polymer–electrolyte fire assessment. LOI (minimum O_2_ to sustain flaming) and SET (mass-normalized after-flame time) are widely used because the procedures are simple, require minimal apparatus, and enable high-throughput screening on thin free-standing or gel-impregnated films. They are formulation-sensitive and give complementary signals of flammability and self-extinction. To avoid bias, one should report thickness/areal mass, imbibed loading, and mounting. Candidates that pass LOI/SET advance to cone calorimetry for quantitative HRR, pHRR, THR, and TTI under fixed flux and to TGA to resolve volatility vs. backbone decomposition with ash-corrected residues.

### 3.2. Battery Abuse Testing

Abuse tests impose credible electrical, thermal, and mechanical faults on live cells to reveal electro-thermal feedbacks, runaway onsets, heat/gas release, and failure modes under realistic boundaries, determining whether materials-level gains in SPEs persist at high state-of-charge and practical limits. Prior experiments show that internal shorts and cathode oxygen release can still drive runaway even with minimized liquid solvent, so abuse testing complements materials metrics and links chemistry to cell- and module-level safety.

#### 3.2.1. Electrical Abuse Tests

An electrical abuse test deliberately exceeds current/voltage limits using external short-circuiting, overcharge, over-discharge, and off-spec charging to read out peak temperature, venting/ignition, gas/ejecta, and post-test health; it is widely used to compare electrolytes and to verify whether SPEs moderate flame/jet without masking high-SOC cathode hazards [49,50].

#### 3.2.2. Thermal Abuse Tests

A thermal abuse test applies external heating (fixed/ramped ovens, shock/cycling, radiant/flame) and near-adiabatic ARC (heat–wait–search) to determine self-heating/runaway onsets, dT/dt, energy/pressure rise, and ignition behavior, chemistry-agnostic benchmarks for liquid vs. SPE/gel systems [51,52].

#### 3.2.3. Mechanical Abuse Tests

A mechanical abuse test challenges structural integrity and emulates internal shorts via crush/impact/vibration and penetration (nail/needle); because nail outcomes depend on geometry/heat-sink effects, controlled internal-short surrogates (slow needle/indentation/FISC) are used for localized, repeatable faults to test whether SPEs truly confine damage and moderate jets/ejecta at high SOCs [53,54,55].

## 4. Safety by Design: Strategies for Fire-Safe Polymer Electrolytes

Polymer electrolytes must carry Li^+^ efficiently while offering minimal fuel and heat feedback to ignition. Thermal runaway shows the hazard: once heat generation exceeds dissipation, volatile species and radical reactions sustain combustion. To counter this, research has developed a set of design routes that tune chemistry, morphology, and architecture while preserving electrochemical stability. We group these routes into eight streams: inorganic fillers that act as heat sinks and form inorganic residues; MOF and COF hosts or fillers that immobilize anions, trap volatiles, and template char; phosphorus systems that quench radicals and catalyze char; halogen strategies that inhibit flame chemistry when allowed; silicon systems that ceramize to silica and reinforce the condensed phase; bio-based chemistries that favor charring; ionic-liquid formulations that lower volatility while maintaining transport; and matrix-engineered layouts, namely sandwich and Janus architecture. Two mechanisms underpin all strategies. Gas-phase action dilutes combustibles and quenches chain-propagating radicals. Condensed-phase action absorbs heat, generates char or ceramic layers, and lengthens diffusion paths for volatiles and oxygen. Effective designs couple both, aiming for improved UL-94 rating, higher limiting oxygen index, lower heat release, and strong battery metrics such as room-temperature conductivity, Li^+^ transference number, stability window, and cycling.

### 4.1. Inorganic Fillers

Inorganic fillers enable condensed-phase fire safety in composite polymer electrolytes by redirecting heat, mass, and ion transport rather than relying on gas-phase quenching. They stiffen and thermally stabilize the matrix, engineer interfacial ion transport, and build barriers that slow ignition and flame spread. Three useful families are minerals, ceramics, and MXenes. Mineral fillers such as metal hydroxides, carbonates, silicas, silicates, layered double hydroxides, and clays absorb heat and release H_2_O or CO_2_, which cools the matrix and dilutes volatiles; the residual inorganic phase forms diffusion barriers. Their polar surfaces lower crystallinity, assist ion-pair dissociation, and immobilize anions, which supports conductivity and increases Li^+^ transference number while reducing melt dripping and smoke. Ceramic fillers including garnet and NASICON provide intrinsic Li^+^ pathways and a thermally robust skeleton; particles or fibers help retain shape at temperature, decouple conduction from segmental motion, immobilize anions at Lewis-acidic sites, physically separate unstable interfaces, and broaden the practical electrochemical window. MXenes such as Ti_3_C_2_T_x_ add two-dimensional barriers with efficient heat spreading; stacked platelets create tortuous diffusion, surface terminations tune crystallinity and anion binding, and high temperature ceramization yields an inorganic shell that further blocks heat and vapor. Effective design depends on surface chemistry, dispersion, orientation, and loading.

#### 4.1.1. Mineral Fillers

**Figure 2:**
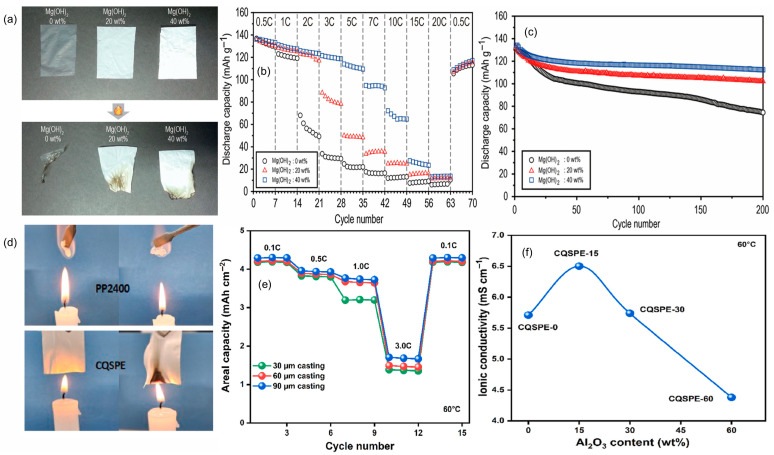
(**a**) Flammability of PVDF-HFP/Mg(OH)_2_ composite electrolytes at different wt% Mg(OH)_2_. (**b**) Rate performance of LiCoO_2_‖graphite cells employing Mg(OH)_2_-filled PVDF-HFP electrolytes under stepped C-rates. (**c**) The 2 C cycling stability over 200 cycles for LiCoO_2_‖graphite cells with Mg(OH)_2_-filled PVDF-HFP electrolytes. (**d**) Pouch-cell-level fire-resistance test of an Al_2_O_3_-PVDF-HFP composite quasi-solid polymer electrolyte (CQSPE). (**e**) Rate capability at 60 °C versus membrane thickness for Al_2_O_3_-CQSPE. (**f**) Ionic conductivity versus temperature and Al_2_O_3_ content for Al_2_O_3_-CQSPE, highlighting the CQSPE-15 formulation. Panels (**a**–**c**) reproduced with permission from Ref. [56] (Elsevier, 2017); panels (**d**–**f**) reproduced with permission from Ref. [57] (Elsevier, 2025).

Mineral fillers provide a strictly inorganic route to flame-retardant polymer electrolytes while preserving ion transport. Instead of relying on external suppressants, the fillers act in the condensed phase, absorbing heat endothermically, releasing non-combustible species in situ, and forming protective inorganic residues that slow ignition and flame spread within the matrix. Because they are embedded where heat and radicals are generated, the safety function is naturally coupled to the ion-transport network. Two case studies show how this strategy improves fire behavior without sacrificing electrochemistry. In PVDF-HFP gels with Mg(OH)_2_, the hydroxide operates as a heat sink and water-releasing species, giving visible self-extinguishing behavior, as demonstrated in Figure 2a. Better electrolyte uptake accompanies higher loading, increasing from 59.2% to 95.5% at 40 wt%, which supports transport under load. At the cell level, the neat gel delivers only 49.2 mAh·g^−1^ at 2 C, whereas 20 and 40 wt% Mg(OH)_2_ reach ≈120 mAh·g^−1^, about 89% of the 0.5 C value, and after 200 cycles at 2 C, the 40 wt% and 20 wt% samples retain 112.4 and 102.1 mAh·g^−1^ compared with 74.4 mAh·g^−1^ for the filler-free control, as reported by Kim et al. The rate response that underpins this outcome is summarized in Figure 2b, and the long-term 2 C stability is shown in Figure 2c. Together, these plots support a practical window below about 40 wt% that improves flame resistance yet maintains mechanical integrity and rate capability [56]. A complementary design uses nanoscale Al_2_O_3_ in a PVDF-HFP composite quasi-solid electrolyte, which demonstrates pouch-cell fire-resistant behavior while retaining high-rate operation, as demonstrated by S.-H. Kim et al. The flame test evidence is shown in Figure 2d, and transport–thickness trade-offs at 60 °C are mapped in Figure 2e, identifying about 60 μm as an effective operating thickness. Ionic conductivity versus temperature is captured in Figure 2f, where the CQSPE-15 formulation reaches 6.5 mS·cm^−1^ at 60 °C. In full cells, a Gr-SiOx‖NCM622 double-stack pouch delivers an initial 1008 mAh, 2.76 mAh·cm^−2^, about 120.6 mAh·g^−1^ at 0.5 C and 1 C, and retains 79.67% after 350 cycles at 60 °C, indicating that condensed-phase protection can be integrated without penalizing kinetics [57]. Complementary mineral chemistries broaden the toolkit. Mesoporous methacrylate-functionalized SiO_2_ raises room-temperature conductivity from 1.1 × 10^−3^ to ≈1.8 × 10^−3^ S·cm^−1^, sustains 142.7 mAh·g^−1^ at 5 C, and suppresses HF after three days at 55 °C, as shown by Shin et al. [58], indicating that porosity and surface functionality can harden gels against thermal and acidic stress while supporting fast discharge. Electrospun PVDF Ca–Al LDH gels combine endothermic water release with char-densifying residues, achieve σ 3.54 × 10^−3^ S·cm^−1^, and operate stably in Li‖LiCoO_2_ with an initial 140.3 mAh·g^−1^ and 99% coulombic efficiency over tens of cycles, with low thermal shrinkage, as reported by Shamitha et al. [59]. As a whole, these studies transform flame mitigation from an external additive into a built-in electrolyte function. The design rules are to choose fillers that provide internal heat sinks or gas-release triggers, couple them with surfaces that improve wetting and interfacial stability, keep loading within a mechanical-integrity window, and employ mesostructured or fibrous scaffolds to maintain continuous ion pathways at elevated temperatures.

#### 4.1.2. Ceramic Families as In Situ Flame Skeletons

The two classes of inorganic solid electrolytes, namely garnet LLZO or LLZTO and NASICON LATP or LAGP, can act as an in situ fire skeleton when they are dispersed in polymer hosts such as PVDF-HFP, PAN, or PEO. These fillers raise heat capacity and thermal conductivity, encourage the formation of an insulating ceramic shell, and suppress evaporation of gel solvents during heating. Lewis-acidic surface sites on these ceramics can immobilize anions and thereby temper parasitic heat from salt decomposition [19]. Recent studies increasingly report direct-flame tests, UL-94 ratings, and limiting oxygen index alongside TGA and DSC, demonstrating real flame delay or self-extinguishing without sacrificing electrochemistry. Together, these mechanisms and test protocols define the common safety framework for the family-by-family comparisons that follow.

##### Garnet Fillers

**Figure 3:**
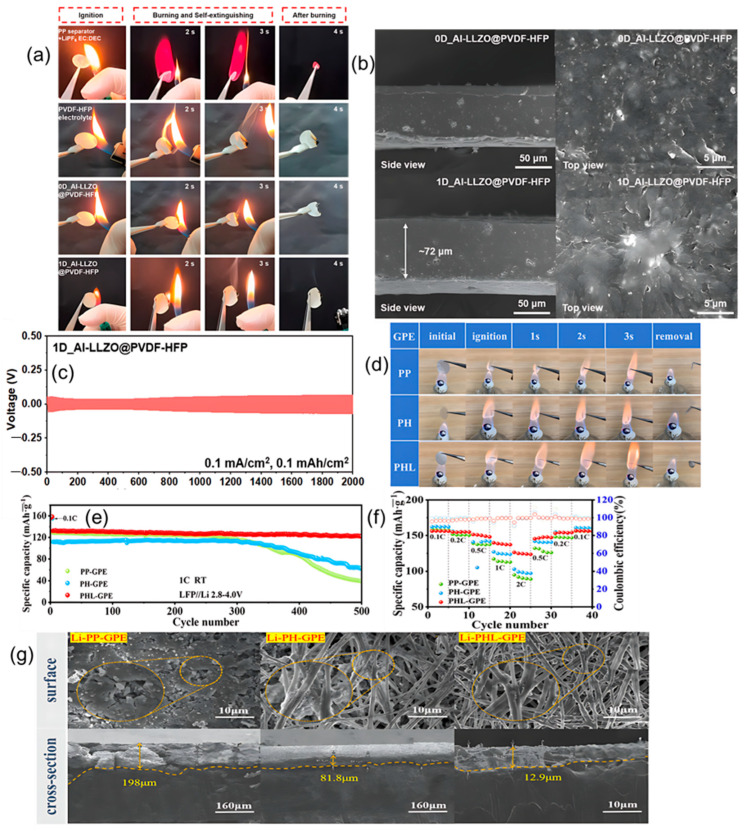
(**a**) Direct-flame photographs comparing PP + LE, neat PVDF-HFP, 0D Al-LLZO@PVDF-HFP, and 1D Al-LLZO@PVDF-HFP. (**b**) Cross-section and top-view SEM morphology of 0D vs. 1D Al-LLZO in PVDF-HFP. (**c**) Symmetric Li‖Li cycling trace for the 1D Al-LLZO@PVDF-HFP electrolyte. (**d**) Film-level torch test of PP-GPE, PH-GPE, and PHL-GPE. (**e**) Full-cell LFP‖Li cycling comparison for PP-GPE, PH-GPE, and PHL-GPE. (**f**) Rate-performance comparison for PP-GPE, PH-GPE, and PHL-GPE. (**g**) SEM images of cycled Li anodes from PP-GPE, PH-GPE, and PHL-GPE cells. Panels (**a**–**c**) reproduced from Ref. [60] (MDPI, 2025); panels (**d**–**g**) reproduced with permission from Ref. [64] (ACS, 2025).

In PVDF-HFP, adding zero-dimensional LLZO particles or one-dimensional LLZO fibers establishes a ceramic scaffold that blocks flames, stiffens the matrix at elevated temperature, and slows heat-driven softening. Im and co-workers make the contrast unambiguous in the direct-flame sequence shown in Figure 3a, where a PP separator soaked with liquid electrolyte ignites and burns without restraint, while PVDF-HFP chars and self-extinguishes, and both 0D and 1D Al-LLZO@PVDF-HFP do not sustain combustion. This film-level safety aligns with electrochemical gains reported for the same series. Relative to neat PVDF-HFP, both Al-LLZO composites not only lower flammability but also widen the electrochemical stability window and raise the critical current density in Li‖Li cells, indicating that flame safety and ion transport can be advanced together. The durability of that coupling appears in Figure 3c, where the 1D composite plates and strips lithium for about 2000 h at 0.1 mA·cm^−2^ with an area capacity of 0.1 mAh·cm^−2^, and in the associated critical current density measurements, where the 1D architecture reaches about 1.3 mA·cm^−2^ compared with about 0.9 mA·cm^−2^ for the 0D composite and about 0.3 mA·cm^−2^ for PVDF-HFP. The structural origin is visible in Figure 3b. Cross-section and top-view SEM morphologies evolve from agglomerated 0D inclusions to a percolated 1D nanowire network embedded in the polymer, with the 1D film presenting a thickness of approximately 72 μm. Postmortem observations then close the loop from morphology to interface. Lithium cycled with the 1D composite shows a markedly flatter and cleaner surface than with PVDF-HFP or 0D fillers, as seen in the right-hand panel of Figure 3b, which is consistent with lower interfacial resistance and dendrite suppression. Complementary electrochemical metrics reinforce this structure–property link. At 30 °C, the 1D composite delivers an ionic conductivity near 1.40 × 10^−4^ S·cm^−1^ and a lithium-ion transference number around 0.75, both higher than the 0D counterpart, and it sustains a stability window close to 4.75 V with a reduced activation energy around 0.20 eV for ion transport [60]. Fang and co-workers demonstrate a complementary dual-filler route in which SiO_2_ aerogel provides a condensed-phase barrier and LLZTO sustains lithium pathways. The composite reaches roughly 1.01 × 10^−3^ S·cm^−1^ and an electrochemical window near 5.0 V at room temperature, supports Li‖Li operation for more than 1500 h at 0.25 mA·cm^−2^, and powers a pouch cell that continues working after folding, cutting, and external short-circuiting [61]. Xu and co-workers extend the same logic to an in situ fire-proof gel initiated by LLZO that combines an ionic conductivity near 1.84 × 10^−3^ S·cm^−1^ at 20 °C with an electrochemical window around 4.75 V, passes cutting and burning tests, and retains 94.08% of the initial discharge capacity after 360 cycles at 0.5 C while maintaining an average coulombic efficiency above 98% [62].

##### NASICON

NASICON composites provide direct and repeatable film-level flame tests and show that condensed-phase protection can coexist with strong transport. LATP-doped PVDF-HFP membranes are explicitly described as flame-retardant while still delivering stable cycling. Transport metrics further support this design space. LATP-doped PVDF-HFP reaches an ionic conductivity of approximately 8.13 × 10^−4^ S·cm^−1^ at 40 °C and a lithium-ion transference number near 0.58, while retaining roughly 90.3% of capacity after 1000 cycles at 0.5 C [63]. Guo and co-workers report a standardized combustion experiment in which PP (polypropylene)-GPE, PH (PVDF-HFP)-GPE, and PHL (PVDF-HFP with LATP)-GPE ignite under a flame yet extinguish themselves in about one second after the flame is removed. The PHL-GPE specimen forms a protective carbonized edge and preserves its disk shape, whereas the PP and PH support curl and deform, as documented in Figure 3d. The same electrolyte architecture couples flame response with decisive electrochemical advantages. In Li‖Li symmetric cells, the PHL-based gel sustains more than 2000 h of cycling at 0.5 mAh·cm^−2^ with small and stable overpotentials near 10 mV, while corresponding cells with PP and PH versions drift to about 200 mV after 700 h and about 300 mV after 1800 h, respectively. Nucleation overpotentials in Li‖Cu tests fall to about 9.7 mV for the PHL-based gel compared with about 58.3 mV for PP and about 66.9 mV for PH, indicating easier and more uniform lithium plating. In full cells at 1 C with a high cathode active mass loading near 11.25 mg cm^−2^, the PHL-based gel delivers an initial discharge capacity around 132.1 mAh·g^−1^ and retains about 122.3 mAh·g^−1^ after 500 cycles, corresponding to a capacity retention near 92.3%; under the same conditions, the PP and PH variants fall to only about 30.1 and 55.9% retention after 500 cycles, which is captured in Figure 3e. The companion rate-capability panel in Figure 3f traces specific capacity from 0.1 C up to 5 C with high coulombic efficiency throughout, and at each rate, the PHL-based gel maintains a clear advantage over PP and PH, reinforcing that the flame-retardant scaffold does not trade away power performance [64]. When these data are read together with electrochemistry and morphology through Figure 3g, the observations converge on a single rule for garnet and NASICON approaches alike. A condensed-phase flame barrier, for example, an inorganic aerogel that resists heat feedback or a carbonized skin that throttles volatiles, should be embedded while percolating inorganic ion-conduction pathways such as LLZO or LLZTO and LATP are maintained. The outcome is self-extinguishing behavior at the film and device levels together with conductivity on the order of 10^−3^ S·cm^−1^ and long-life cycling in lithium-metal cells.

#### 4.1.3. MXenes

**Figure 4:**
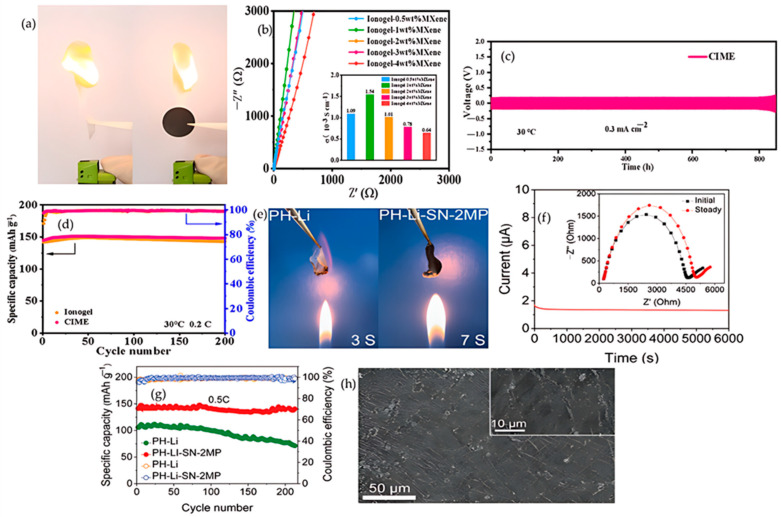
(**a**) Torch/self-extinguishing photographs of a PVDF-HFP ionogel containing MXene exhibiting fire-resistant behavior. (**b**) Nyquist plots and ionic-conductivity analysis versus MXene loading for a PVDF-HFP–ionic-liquid ionogel at room temperature. (**c**) Symmetric Li‖Li galvanostatic cycling demonstrating stable plating/stripping. (**d**) LiFePO_4_‖Li full-cell cycling showing stable capacity retention. (**e**) Flame photographs comparing PH-Li (control) and PH-Li-SN-2MP with mPEG-grafted MXene, indicating enhanced combustion resistance. (**f**) Bruce–Vincent polarization and EIS determining lithium-ion transference number and room-temperature conductivity for the MXene-engineered electrolyte. (**g**) NCM‖Li full-cell cycling at a constant rate showing durable performance. (**h**) SEM images of Li anodes after cycling illustrating more uniform deposition with the MXene-engineered electrolyte. Panels (**a**–**d**) reproduced with permission from Ref. [66] (ACS, 2023); panels (**e**–**h**) reproduced from Ref. [69] (Wiley-VCH, 2025).

From a safety perspective, MXenes, typified by Ti_3_C_2_T_x_, are promising two-dimensional fillers for safety by electrolyte design. Beyond boosting ion transport, stacked platelets lengthen diffusion paths for combustibles, high in-plane thermal conductivity damps local hot spots, and Ti-O or Ti-F terminations can ceramize to TiO_2_ or LiF under heat, forming an inorganic barrier that curbs heat and mass transfer. These principles are already established for polymer systems and are translatable to polymer electrolytes for lithium batteries [65]. This promise is borne out experimentally in a PVDF-HFP composite ionogel containing MXene, where Tang and co-workers report room-temperature conductivity of 1.54 × 10^−3^ S·cm^−1^ at 25–30 °C as shown in Figure 4b, Li‖Li cycling for ≈800 h at 0.3 mA·cm^−2^ with 190 mV overpotential as illustrated in Figure 4c, and LiFePO_4_‖Li capacity retention of 97.8% after 200 cycles at 0.2 C and 30 °C as shown in Figure 4d; consistent with condensed-phase shielding by the platelets, the electrolyte is described as a fireproof ionogel polymer electrolyte, and the protective architecture is shown in Figure 4a [66]. Within the broader incombustible-electrolyte framework, these condensed-phase protections should be evaluated in lockstep with electrochemistry using self-extinguishing demonstrations, the limiting oxygen index, and UL-94 so that flame behavior and battery performance appear within one coherent system-level narrative; in parallel, MXene regulates the lithium-metal interface by anchoring anions, lowering host crystallinity, and homogenizing the local field, which stiffens the matrix against dendrite penetration and suppresses hot-spot formation that can precipitate thermal runaway [65,67], while co-reporting flame tests together with transport and cycling data makes the safety and performance trade space explicit and comparable across studies [68]. Extending these ideas, surface engineering offers a complementary lever: He and co-workers show that polyethylene glycol-decorated MXene embedded in PVDF-HFP or succinonitrile solid polymer electrolytes delivers 1.49 × 10^−4^ S·cm^−1^ at 30 °C and a lithium transference number near 0.6 as shown in Figure 4f, exhibits rapid ignition for the control at 3 s with no sustained combustion for the engineered film over 7 s as shown in Figure 4e, produces smoother lithium deposition as shown in Figure 4h, and sustains NCM performance around 140.5 mAh·g^−1^ with 99.6% retention for >200 cycles at 0.5 C as shown in Figure 4g. Taken together with the preceding metrics, sustained Li‖Li plating and stripping for >2100 h at 0.1 mA·cm^−2^ corroborates electrochemical safety and is consistent with the condensed-phase protections described above [69].

Fire safety in polymer electrolytes can rise without sacrificing room-temperature transport when the structure is tuned along three complementary routes: mineral fillers, ceramic fillers, and MXene. Mineral fillers curb flammability and stabilize dimensions, yet they require mesoporosity and moderate loading to avoid tortuosity and loss of uptake. Ceramic fillers add intrinsic lithium-ion pathways, lower polymer crystallinity, and steady interfaces when used at an optimal fraction in porous, surface-treated hosts. MXene, used as dispersed sheets, interlayers, or within ionogels, enhances conduction and moderates lithium nucleation when loading stays below electronic percolation and oxidation is controlled. The common pattern is moderate filler content, porous architecture, and functional surfaces, with fire tests and electrochemistry verified on the same formulation at room and elevated temperatures. For a synoptic overview, Table 1 provides a concise, side-by-side synthesis of the key performance metrics, advantages and limitations, trade-offs, and design levers across the three systems.

### 4.2. Crystalline Porous Frameworks (MOFs and COFs)

Crystalline porous frameworks connect molecular building units into periodic lattices with uniform pores and programmable surface chemistry. In solid polymer electrolytes (SPEs), these reticular materials regulate salt dissociation, reduce excess polymer crystallinity, and create continuous Li^+^ pathways; under heat, they enable condensed-phase char formation, radical quenching, and barrier residues that curb ignition and heat release. Safety and transport need to be evaluated together using limiting oxygen index or UL-94, cone or micro-calorimetry outputs, ionic conductivity, Li^+^ transference number, oxidative stability, symmetric-cell lifetime, and full-cell retention [70,71,72].

#### 4.2.1. Metal–Organic Frameworks (MOFs)

**Figure 5:**
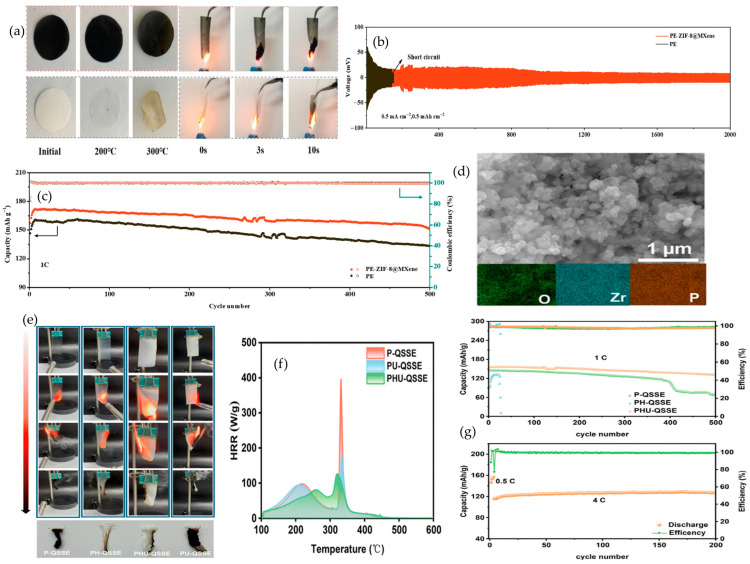
(**a**) Thermal shape retention at 200 and 300 °C and direct-flame test images of the PE–ZIF-8@MXene composite membrane, showing rapid self-extinguishing. (**b**) Symmetric Li‖Li cycling at 0.5 mA cm^−2^. (**c**) LiFePO_4_‖Li full-cell cycling. (**d**) SEM and EDS mapping evidencing confinement of hexachlorocyclotriphosphazene (HCCP) within UiO-66 (HCCP@UiO-66) in a PVDF-HFP quasi-solid membrane. (**e**) Direct-flame photographs showing rapid self-quenching of the HCCP@UiO-66-based membrane. (**f**) Micro-combustion calorimetry curves demonstrating a reduced peak heat-release rate for the MOF-containing quasi-solid electrolyte. (**g**) Full-cell endurance combining 1 C cycling for 500 cycles and 4 C cycling for 200 cycles. Panels (**a**–**c**) reproduced with permission from Ref. [75] (Elsevier, 2022); panels (**d**–**g**) reproduced with permission from Ref. [76] (Royal Society of Chemistry, 2025).

Lewis-acidic nodes and polar pore walls immobilize anions and template Li^+^ percolation, while coordinatively active metals and heteroatom-rich linkers promote carbonization and intercept flame radicals; porous lattices can also confine plasticizers or organophosphates so they do not volatilize under abuse. The combined effect is lower peak heat release and smoother Li-metal current distribution without sacrificing room-temperature transport, consistent with the broader observation that neat PEO is easily ignitable with an LOI near 18% whereas representative MOF-SPEs self-extinguish and deliver double-digit pHRR cuts under calorimetry [19,72]. In the same vein, Jiang et al. report that a defective CeO_2_@Ce-BTC nanorod reinforcement in PVDF-HFP (1 wt%) suppresses PVDF-HFP crystallinity from 3.8% to 1.7% and lifts σ to 2.5 × 10^−4^ S·cm^−1^ at 25 °C (rising to 7.6 × 10^−4^ S·cm^−1^ in a gelled PC system), while pushing tLi^+^ to 0.78 via Lewis-acidic CeO_2_/MOF sites that immobilize TFSI^−^; LSV/CICC indicates an oxidative limit around 4.5 V vs. Li/Li^+^ (approaching 4.8 V depending on configuration), and thermal/combustion tests show shape retention to 300 °C and low flammability with self-quenching after a 3 s flame exposure. In Li‖LFP full cells, the same composite delivers 161.9 mAh·g^−1^ initially at 0.5 C and retains 103.8 mAh·g^−1^ after 100 cycles with 99% coulombic efficiency, consistent with anion-regulated transport and char-assisted heat shielding [73]. Sun et al. [74] establish this coupling in PEO/LiTFSI with activated HKUST-1, where Cu nodes convert to CuO during pyrolysis to seed a sealing carbonaceous char and the porous lattice immobilizes TFSI^−^ while depressing PEO crystallinity; the electrolyte therefore cuts pHRR by about 42% yet preserves σ near 3.5 × 10^−4^ S·cm^−1^ at 50 °C and 2.4 × 10^−3^ S·cm^−1^ at 80 °C, extends the oxidative window to about 4.71 V, raises tLi^+^ from roughly 0.23 to roughly 0.38, self-extinguishes in vertical-flame checks, and sustains about 160 mAh·g^−1^ at 0.1 C with approximately 99% coulombic efficiency while Li‖Li remains stable for about 240 h at 0.1 mA·cm^−2^. Building on the same mechanism, Zhao et al. [75] incorporated MOF porosity with a two-dimensional lamellar structure: ZIF-8 provides Lewis-acidic apertures and short ion corridors, and MXene spreads heat and blunts dendrite pressure. The composite retains geometry at 200 to 300 °C and self-extinguishes under a direct flame as shown in Figure 5a; then, it carries those safety traits into operation with σ near 4.4 mS·cm^−1^ and tLi^+^ near 0.76, symmetric Li‖Li cycling for roughly 2000 h with low polarization in Figure 5b, and Li‖LFP capacity retention around 89.6% over 500 cycles, as shown in Figure 5c, a coherent picture of fast ion highways through MOF pores supported by multi-scale barriers to heat and mass transfer. Li et al. [76] internalize the flame-retardant chemistry by confining a phosphazene within UiO-66 and dispersing the MOF into a PVDF-HFP quasi-solid; SEM and EDS reveal encapsulation in Figure 5d, direct-flame images and micro-combustion calorimetry report rapid self-quenching and a markedly reduced heat-release rate in Figure 5e,f, and the same membrane preserves oxidative headroom near 4.9 V and room-temperature transport with tLi^+^ around 0.59 while delivering 156 mAh·g^−1^ with 84.6% retention after 500 cycles at 1 C and stable operation for 200 cycles at 4 C, as illustrate in Figure 5g. Completing the progression, Zheng et al. [77] distribute these functions across a 3D PVDF-HFP framework reinforced with Li-ionic-liquid-loaded ZIF-8, which lifts the limiting oxygen index to about 20.5% with a clear tendency to self-extinguish, maintains shape under heat, and provides percolated Li^+^ pathways that yield about 2.89 × 10^−4^ S·cm^−1^ at 25 °C and 0.91 × 10^−3^ S·cm^−1^ at 55 °C with a transference number near 0.40 and an oxidative window up to roughly 4.8 V at 55 °C, while Li‖Li runs stably for more than 1000 h at 0.1 mA cm^−2^ and LiFePO_4_ approaches 160 mAh·g^−1^. Considered together, the four systems outline a single design law rather than separate case studies: use polar channels and redox-active nodes to design the flame and the ions simultaneously and then verify the coupling by reading thermal abuse images and HRR curves alongside long-horizon Li‖Li and Li‖LFP data.

In summary, crystalline porous frameworks extend polymer electrolytes with ordered, programmable ion pathways while strengthening fire performance and mechanics. MOFs contribute high surface area and tunable chemistry for anion immobilization and flame-retardant hosting but risk moisture sensitivity and pore blocking at excessive loading. For more information, Table 2 summarizes the pros, cons, core trade-offs, and design levers for COFs in polymer electrolytes.

#### 4.2.2. Covalent Organic Frameworks (COFs)

**Figure 6:**
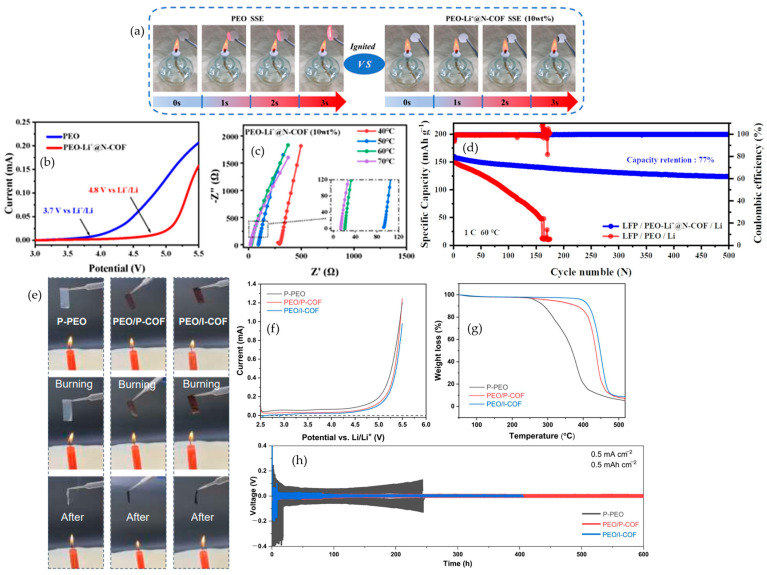
(**a**) Direct-flame test images showing that neat PEO ignites and drips, whereas a 10 wt% N-COF composite membrane self-extinguishes. (**b**) Linear-sweep voltammetry of the N-COF–reinforced PEO electrolyte. (**c**) Temperature-dependent EIS and ionic conductivity for N-COF-reinforced PEO membranes. (**d**) LiFePO_4_‖Li full-cell cycling performance at 1 C and 60 °C. (**e**) Flame-exposure photographs for P-PEO and COF-reinforced PEO membranes. (**f**) Linear-sweep voltammetry of P-PEO, PEO/P-COF, and PEO/I-COF. (**g**) Thermogravimetric analysis showing decomposition onset shifted from 250 °C (P-PEO) to 400 °C (COF composites). (**h**) Symmetric Li‖Li cycling at room temperature for PEO/P-COF and PEO/I-COF electrolytes. Panels (**a**–**d**) reproduced with permission from Ref. [79] (Elsevier, 2024); panels (**e**–**h**) reproduced with permission from Ref. [80] (Elsevier, 2024).

COFs integrate fire safety with electrochemistry. Thermal rearrangement of imide to benzoxazole releases CO_2_ that dilutes flammable vapors, while the aromatic backbone densifies into graphitizable char, sealing the membrane. MCC shows low peak and total heat release, and direct-flame tests show self-extinguishing. When open channels host PEG and lithium salt, the electrolyte maintains room-temperature transport with near single-ion character and a wider oxidative window, advancing safety and rate [78]. Shen et al. show that, in PEO, a nitrogen-rich COF lattice both suppresses combustion and steadies the interfaces. Figure 6a shows neat PEO ignites and drips in 3 s, while a nitrogen-rich COF membrane at 10 wt% does not sustain burning and darkens by in situ carbonization. As shown in Figure 6b, anodic stability widens from 3.7 V versus Li/Li^+^ for the polymer baseline to 4.8 V for the COF composite. In Figure 6c, LFP‖Li cycled between 2.4 and 3.8 V at 1 C and 60 °C begins near 155.8 mAh·g^−1^ and still delivers 121.7 mAh·g^−1^ after 500 cycles, 77% retention with CE 100%. As shown in Figure 6d, Nyquist arcs contract from 40 to 70 °C. At 60 °C, the ionic conductivity reaches 2.4 × 10^−4^ S·cm^−1^ for 10 wt% N-COF compared with 1.0 × 10^−5^ S cm^−1^ for neat PEO, and 5 wt% and 20 wt% give 7.2 × 10^−5^ and 2.2 × 10^−4^ S·cm^−1^, reflecting anion immobilization and oriented Li^+^ pathways [79]. Saleem et al. report the same principle. Pyrazine and imine linkages build protective char, widen stability, and sustain lithium cycling. As shown in Figure 6e, the polymer host ignites and deforms, while pyrazine- and imine-linked COF membranes resist sustained combustion and are darkened by protective char. In Figure 6g, the decomposition onset shifts from 250 °C for the polymer baseline to 400 °C for PEO/P-COF and PEO/I-COF. In Figure 6f, the oxidative window expands to 4.8 V for pyrazine and 5.0 V for imine vs. 4.6 V for the baseline. In Figure 6h, at room temperature, a current density of 0.5 mA cm^−2^, and an area capacity of 0.5 mAh·cm^−2^, the pyrazine composite sustains lithium plating and stripping for 600 h with low polarization, and the imine composite remains stable for 400 h, while the polymer polarizes more and deteriorates earlier [80]. Together, these outcomes support a unified design-the-flame/design-the-ions strategy. Aromatic backbones and strong linkages yield self-sealing char and self-extinguishing. Ionophilic channels immobilize anions, raise lithium transference, and suppress exothermic side reactions. Two-dimensional networks in PEO stabilize interfaces and widen the voltage window. To substantiate safety and speed, UL-94 or vertical-flame testing can be paired with TGA, MCC, or cone calorimetry, plus metrics for ionic conductivity, transference number, critical current density, extended Li‖Li cycling, and long-cycle full-cell performance, interpreted in a mechanistic frame [79,81,82]. The material platform brings clear advantages such as low density relative to inorganic fillers, configurable linkages that enable high char yields or gas-phase dilution, intrinsically aligned nanochannels that decouple conductivity from polymer segmental motion, and the possibility of building single-ion behavior into pore walls. Remaining challenges include scalable synthesis of highly crystalline frameworks, dispersion and exfoliation at useful loadings, sensitivity to moisture or residual catalysts, and composition drift in quasi-solids if the confined solvent redistributes; room-temperature or in situ routes and surface-grafting tactics are promising ways to improve manufacturability and compatibility while preserving flame performance.

In summary, crystalline porous frameworks extend polymer electrolytes with ordered, programmable ion pathways while strengthening fire performance and mechanics. COFs provide lightweight, nitrogen-rich channels with strong thermal stability yet can suffer from modest intrinsic conductivity and over-crystallization. Effective designs keep loading moderate, preserve open and well-wetted pores, and use surface functionalization or thin interlayers to limit interfacial losses. For a concise comparison, Table 3 summarizes the pros, cons, core trade-offs, and design levers for COFs in polymer electrolytes.

### 4.3. Phosphorus-Based Additives

**Figure 7:**
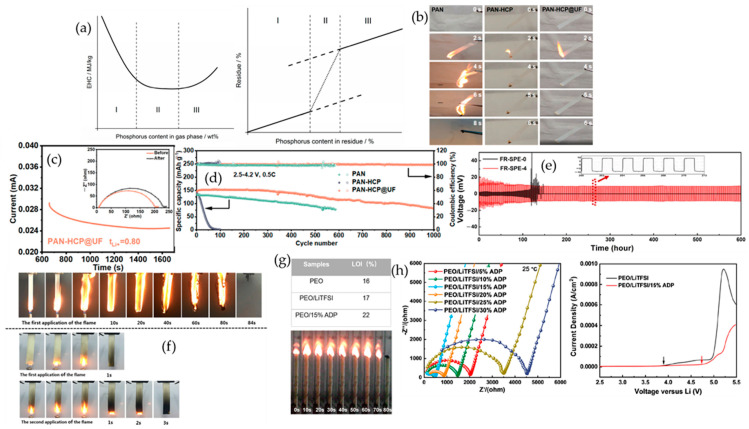
(**a**) Dual-pathway phosphorus mechanism in solid polymer electrolytes: left—EHC decreases with increasing gas-phase phosphorus (radical scavenging); right—residue rises with phosphorus retained in the condensed phase (polyphosphate barrier/intumescence). (**b**) Direct-flame images showing that an HCP@UF microcapsule-reinforced solid polymer electrolyte self-extinguishes in 2 s with LOI = 28%. (**c**) Lithium-ion transference number for the HCP@UF system. (**d**) LiFePO_4_‖Li full-cell cycling at 0.5 C for the HCP@UF system. (**e**) Symmetric Li‖Li plating–stripping stability for phosphorus-containing polyurethane-acrylate membrane. (**f**) Direct-flame and UL-94 comparison: phosphorus-free membrane (top) vs. phosphorus-containing polyurethane-acrylate membrane (bottom). (**g**) Flame-test sequence and limiting-oxygen-index summary for an ADP/PEO electrolyte. (**h**) Temperature-dependent ionic conductivity and linear-sweep voltammetry window for ADP/PEO. Panel (**a**) reproduced from Ref. [84] (MDPI, 2017); panels (**b**–**d**) reproduced with permission from Ref. [88] (ACS, 2024); panels (**e**,**f**) reproduced with permission from Ref. [91] (ACS, 2022); panels (**g**,**h**) reproduced with permission from Ref. [93] (ACS, 2021). Direct-flame and UL-94 comparison: phosphorus-free membrane (top) vs. phosphorus-containing polyurethane-acrylate membrane (bottom).

Phosphorus additives protect solid polymer electrolytes through a coupled response in which condensed-phase chemistry predominates while a controllable gas-phase contribution supports ignition resistance. Upon heating, phosphate-forming species generate phosphoric and pyrophosphoric acids that catalyze dehydration and crosslinking, raise melt viscosity, advance aromatization, and build a cohesive poly(meta)phosphate barrier that throttles volatile release and heat feedback. Figure 7a visualizes the mechanism in a single schematic by integrating two elements. The first element shows residue formation intensifying as phosphorus is retained in the condensed phase, marking the transition to a robust poly(meta)phosphate barrier that can intumesce when expanding gases are available and thereby further impede heat and mass transfer. The second element depicts the gas-phase contribution in which thermal scission releases PO· and HPO· radicals that scavenge H· and OH·, shorten chain branching, and lower flame temperature, which corresponds to a decline in effective heat of combustion as more phosphorus enters the flame. The balance between these pathways is tuned by oxidation state, substituents, volatility, and host functionality, and for battery-grade solid polymer electrolytes, covalent immobilization or abuse-triggered release preserves barrier efficacy through cycling while limiting leaching and plasticization [83,84,85].

#### 4.3.1. Cyclophosphazenes

Cyclophosphazene motifs perform best when their phosphorus functionality is fixed inside the electrolyte so that condensed-phase chemistry can activate in situ under abuse. Zhou et al. show that a UV-photopolymerized cyclophosphazene network sustains thermal stability near 300 °C, ionic conductivity around 3.7 × 10^−4^ S·cm^−1^ at 60 °C, and Li‖LiFePO_4_ capacities near 130 mAh·g^−1^ at 0.1 C with coulombic efficiency close to 99%; flame tests in the same work indicate a phosphate-rich char as the operative defense [86]. Zuo et al. advance the architecture by epoxy–amine anchoring of hexa-substituted rings, keeping conductivity near 1 × 10^−4^ S·cm^−1^ at 30 °C while lifting T_5_% to about 368 °C and visibly suppressing burning relative to PEO, which is consistent with a stiffer condensed-phase scaffold [87]. The causal thread from flame behavior to transport becomes explicit in the microcapsule study of Zhang et al. Figure 7b captures self-extinguishing in roughly two seconds together with a limiting oxygen index of 28%. Figure 7c establishes a lithium-ion transference number of 0.80. Figure 7d shows Li‖LiFePO_4_ retaining 88.8% after 500 cycles at 0.5 C, aligning barrier formation, cation selectivity, and long-cycle stability within one formulation [88]. Tu et al. then demonstrates that locking a cyclophosphazene derivative inside the device by in situ gelation yields nonflammable gels that self-extinguish in under five seconds, maintain an electrochemical window above 5.0 V versus Li/Li^+^, and sustain 200 cycles at 0.2 C, which confirms that ignition resistance can coexist with practical operation [89].

#### 4.3.2. Organophosphates

Organophosphate chemistry allows volatility, acid yield, and char cohesion to be tuned so the condensed-phase response dominates while a measured gas-phase contribution trims ignition. Olmedo-Martínez et al. embed phosphate backbones into a polyphosphoester that conducts lithium near 2.0 × 10^−4^ S·cm^−1^ at 70 °C with a transference number around 0.26. Cells at 70 °C retain about 80% after 100 cycles with coulombic efficiency above 98%, and micro-calorimetry verifies reduced heat release compared with PEO [90]. The value of covalent fixation is clearest in the UV-curable platform of Lv et al. Figure 7f documents direct-flame self-quenching that underpins a UL-94 V-0 rating as the grafting degree approaches 39%. Figure 7e extends the electrochemical argument with more than 600 h of stable Li‖Li plating and stripping at 0.1 mA cm^−2^ while the membrane maintains conductivity near 2.66 × 10^−4^ S·cm^−1^ at 25 °C, a stability window around 5.0 V, a transference number of 0.55, and Li‖LiFePO_4_ performance of 144.6 mAh·g^−1^ after 100 cycles at 0.2 C together with 107.4 mAh·g^−1^ at 5 C [91]. In a PEO matrix, Liu et al. showed that a polyphosphonate (PBMP) delivers UL-94 V-0 with a limiting oxygen index of 25.6% at 3 wt% P. Micro-combustion calorimetry recorded ≈21% lower peak heat-release rate and ≈15% lower total heat release, with the heat-release peak shifted ≈23 °C higher—evidence of protective char formation. Electrochemically, PEO/PBMP/LiTFSI preserved a 4.0 V stability window and reached 1.25 × 10^−5^ S·cm^−1^ at 25 °C (4.7 × 10^−5^ S·cm^−1^ at 55 °C). Coin cells (LFP‖Li, 55 °C) sustained 150 mAh·g^−1^ from cycle 20 to 100 at 0.02 C, indicating flame-retardant design can coexist with robust transport and cycling [92]. The architectural lesson is that once the phosphate is grafted into the host, the barrier chemistry remains available at the very interface where heat and radicals originate without compromising the ion-transport network required for routine cycling.

#### 4.3.3. Metal Phosphinates

Metal phosphinates such as aluminum diethyl phosphinate (ADP) are non-volatile organophosphorus salts whose protection is inherently condensed-phase, building cohesive phosphate-rich residues that resist heat and mass feedback. Han et al. illustrate this with a direct blend into PEO. Figure 7g presents a flame-test series together with a limiting oxygen index that rises to 22% for PEO containing 15% ADP compared with 16 to 17% for controls, which signals a move toward self-extinguishing behavior. Figure 7h assembles the transport picture with Arrhenius conductivity trends and linear-sweep voltammetry approaching a stability window near 5.0 V versus Li/Li^+^, aligning safety with usable ion transport. The same composition validates at device scale as LiFePO_4_‖Li at 60 °C sustains about 123.2 mAh·g^−1^ at 1 C after 1000 cycles with coulombic efficiency close to 99.95%, a result attributed to an Al, P-rich interphase that stabilizes lithium in parallel with char formation [93]. Extending the concept, Zhang et al. microencapsulate the phosphinate prior to blending and raise the limiting oxygen index to 27% while preserving rate capability and lifetime near 103 mAh·g^−1^ at 1 C for roughly 800 cycles, which indicates that metered release and improved dispersion can deliver stronger fire performance without taxing transport [94]. Read as a sequence, these panels show how immobilized phosphorus strategies deliver self-extinguishing behavior and measurable safety gains while keeping the electrochemical core intact. Cyclophosphazene networks and capsules couple rapid flame quenching with high transference number and durable full-cell cycling. Grafted organophosphates reach UL-94 V-0 while sustaining long-term lithium plating and stripping and practical power response. Metal phosphinates bias protection to the condensed phase and, with or without encapsulation, support a wide electrochemical window and long life. The unifying rule is to place phosphorus exactly where it must act, inside the electrolyte matrix, so barrier formation and radical moderation trigger at the point of hazard without sacrificing the pathways that carry lithium.

To distill the essentials, phosphorus additives act mainly in the condensed phase through char formation and acid-catalyzed protection, with PO radical chemistry as a secondary gas-phase route. At moderate loading and when immobilized by tethering or formed in situ, they enable interphase engineering with a P-rich or metal phosphate SEI and strengthen thermal and mechanical robustness. Overloading, hydrolytic sensitivity, migration, or poor voltage matching can increase interfacial impedance and weaken ion transport. Key considerations are synthesized in Table 4.

### 4.4. Halogen-Based Additives for Flame Suppression in Solid Polymer Electrolytes

Halogenated additives act first in the gas phase. When heated, they release hydrogen halides and halogen radicals that rapidly consume H· and OH·, which lowers the heat-release rate and interrupts the radical chain that sustains combustion [95]. Intrinsic gas-phase inhibition generally follows F < Cl < Br ≤ I. In practice, solid polymer electrolytes are selected not only for inhibition strength but also for ionic transport and interfacial stability, so fluorine- and bromine-containing systems dominate truly solid matrices, whereas chlorine and iodine appear far less often or in latent-release and hybrid constructs [65].

#### 4.4.1. Fluorine (F) Additives

**Figure 8:**
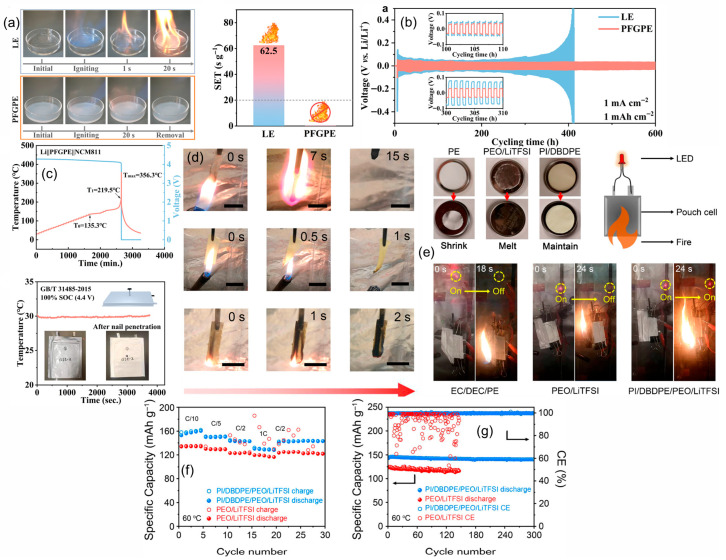
(**a**) Direct-flame images showing an in situ perfluorinated gel self-extinguishing and SET values comparison of LE and PFGPE. (**b**) Symmetric Li‖Li plating–stripping profile. (**c**) Accelerating rate calorimetry and safety test results. (**d**) Direct-flame sequence of a PI/DBDPE/PEO/LiTFSI laminate showing self-quenching. (**e**) Pouch-cell torch test with a brominated laminate. (**f**) Rate capability of Li/PEO/LiTFSI/LFP and Li/PI/DBDPE/PEO/LiTFSI/LFP cells tested at 60 °C. (**g**) Long-term cycling at C/2 for the same cells at 60 °C. Panels (**a**–**c**) reproduced with permission from Ref. [97] (Elsevier, 2024); panels (**d**–**g**) reproduced with permission from Ref. [101] (ACS, 2020).

The most persuasive fluorine strategies treat fire safety and interfacial design as the same problem. In the in situ perfluorinated gel reported by Yang et al., continuous-flame images and the self-extinguishing metric demonstrate a complete suppression of sustained burning, with the value falling from 62.5 s·g^−1^ for the liquid electrolyte to 0 s·g^−1^ for the gel; this behavior is shown in Figure 8a. The same chemistry stabilizes lithium plating and stripping. Figure 8b shows that symmetric Li‖Li cells persist for more than 300 h at 1 mA·cm^−2^ and 1 mAh·cm^−2^, whereas the liquid fails near 110 h, indicating that the non-ignitable state coincides with a quieter, stronger interface. Abuse testing extends the argument from bench to device. Figure 8c records the onset temperatures in accelerating-rate calorimetry shifting to 135.3 °C and 219.5 °C, the peak temperature dropping to 356.3 °C, and the pouch cell passing GB/T 31485 [96] heating and nail-penetration protocols, which together indicate that condensed-phase control of volatiles and a LiF-rich interphase delay heat feedback and interrupt runaway at scale [97]. That mechanistic picture holds across formats. Hu et al. place mesoporous AlF_3_ inside PEO to bias interphases toward LiF while preserving pathways for transport; the lithium-ion transference number rises to about 0.67 at 60 °C, and thin laminated Li‖SPE‖FeF_3_ cells evolve from roughly 600 to roughly 200 mAh·g^−1^ over 900 cycles, showing that fluorinated fillers can harden the matrix and sustain capacity simultaneously [98]. Fluorinated solids with intrinsic self-healing reported by Wang et al. remain nonflammable, maintain a stability window near 4.9 V, and deliver around 145 mAh·g^−1^ at 0.2 C with approximately 82% retention, which shows that recoverable mechanics can coexist with safety in a truly solid matrix [99]. A separate fluorinated gel from Wang et al. reconnects nonflammability with demanding lithium-metal and high-voltage operation by combining zero self-extinguishing time with more than 2000 h of Li‖Li stability and extended NCM811 cycling near 4.5 V [100]. Read together, these results show why fluorination succeeds: when the functionality is embedded as a network or a mesostructured filler, the same LiF-mediated interphase that limits parasitic reactions also limits fuel generation and heat release, so ignition resistance, transport metrics, voltage stability, and pouch-cell durability rise in concert.

#### 4.4.2. Bromine (Br) Additives

Bromine addresses combustion in the gas phase, but the exemplary studies make its radical quenching work only when the halogen is architecturally anchored so mobility is not compromised. Cui et al. immobilize decabromodiphenyl ethane (DPDPE) in a porous polyimide scaffold imbibed with PEO/LiTFSI and capture rapid self-quenching under a direct flame; the sequence in Figure 8d shows no sustained burning within about two seconds. The same laminate demonstrates device-level resilience. Figure 8e shows a pouch-cell torch test in which the LED remains lit for the brominated laminate while the liquid carbonate control and the neat PEO/LiTFSI film fail, which ties gas-phase inhibition directly to practical abuse tolerance. Electrochemistry remains intact. Figure 8f,g report rate performance at 60 °C retaining about 163, 152, 143, and 131 mAh·g^−1^ from C/10 to 1 C and long-term cycling at C/2 that stays flat for roughly 300 cycles with near-unity coulombic efficiency, indicating that strong quenching is achieved at a dose low enough to preserve ion pathways [101]. The chemistry can also be steered to benefit interfaces at high voltage. Zhou et al. show that charging toward about 4.5 V oxidizes a brominated inhibitor to generate LiBr in situ, which templates an organic-rich, LiBr-containing interphase on NCM811 while flame-retardant behavior is maintained; the cells deliver around 151 mAh·g^−1^ with about 83% retention and an average 99.7% coulombic efficiency over 150 cycles [102]. When bromine is covalently integrated, as in the polyurethane solid electrolyte of Wu et al., vertical-burn trials record no sustained burning, the stability window extends to roughly 5.1 V, symmetric Li‖Li runs for more than 2100 h at 0.2 mA·cm^−2^ and 0.2 mAh·cm^−2^, and layered-oxide full cells retain capacity over hundreds of cycles [103]. The design rule that emerges is to immobilize bromine so gas-phase inhibition is strong at modest loading and then to use LiBr-mediated interphases to reconcile fire safety with high-voltage cycling, ensuring that flame metrics and electrochemistry are demonstrated in the same construct.

Briefly stated, halogen additives suppress flames primarily via gas-phase radical quenching. Fluorinated designs can widen the oxidative window and stabilize interphases, while brominated routes provide strong suppression but require careful control of corrosion and byproducts. Benefits peak at moderate loading with tethered or in situ systems, whereas excessive content or inadequate passivation can penalize transport and accelerate aging. A concise synopsis is provided in Table 5.

### 4.5. Silicon-Based Additives

Solid polymer electrolytes are pursued to replace flammable liquid electrolytes in lithium-ion batteries, yet most host matrices, such as PEO, PAN, and PVDF, remain combustible, so integrating flame-retardant additives is essential. Silicon-containing systems are attractive because organosilicon moieties convert under heat to a protective silica layer that insulates the substrate from oxygen and thermal feedback, suppressing flame propagation while raising thermal stability. Many silicon-bearing polymers also exhibit comparatively low heat release and higher thermal tolerance, although incorporating siloxane or silica segments can lower polarity and weaken Li^+^ coordination, which may depress ionic conductivity if compositions are not judiciously designed. The dominant action is condensed-phase: Si-containing groups ceramize to SiO_2_ or silicates that build a stable barrier, and in hybrids with phosphorus, a well-documented synergy emerges in which phosphorus promotes dehydrative char and radical quenching while silicon stabilizes and reinforces that char so peak heat release and flame spread fall at lower total additive loadings [19,20,72]. The following subsections detail representative classes used to realize these mechanisms in practice.

#### 4.5.1. Inorganic Silicon Additives

Across inorganic silicon additives, the safety function is anchored in the condensed phase. Thermally stable and non-combustible species such as SiO_2_ nanoparticles, silicate clays, and halloysite nanotubes build ceramic-rich barriers that throttle heat feedback and oxygen access while limiting fuel volatilization. In PEO/silica nanocomposites, ignition shifts later, decomposition occurs at higher temperature, and char residue increases relative to neat PEO, which is consistent with a silica-reinforced glaze that suppresses flame spread [19,20,72]. Pongsuk and Pumchusak show that halloysite nanotubes, with a silica-rich exterior and an alumina lumen, assemble heat-resistant networks that catalyze char formation and slow heat and mass transport; in a PEO-Li salt electrolyte containing 5 wt% halloysite, ignition is delayed to about 30 s under direct flame, and the strip self-extinguishes at about 40 s after removing the heat source [104]. Nanosilica that is well dispersed hardens the matrix while depressing PEO crystallinity and behaves as a solid plasticizer that preserves the 10^−4^ S·cm^−1^ conductivity regime at room or modestly elevated temperature; it also widens the anodic window and raises Li^+^ transference through anion interactions with surface silanols. Li et al. report that negatively charged laponite platelets stiffen films and reorganize Li^+^ coordination; a PEO–laponite composite exhibits σ of 1.1 × 10^−3^ S·cm^−1^ at 60 °C and enables LFP‖Li cells to cycle beyond 800 cycles at 1 C and 60 °C with about 99.9% coulombic efficiency, which shows that safety gains do not preclude durable, high-rate operation [105]. Luo et al. find that two-dimensional silica nanosheets retain about 10^−4^ S·cm^−1^ near 30 °C while increasing modulus and thermal tolerance, and this matches observations of a rapid SiO_2_ glaze under flame and aligns with stable Li plating, a transference number near 0.34, and full-cell capacities near 159 mAh·g^−1^ at 0.2 C with strong retention [106]. Wang et al. demonstrate that an in situ TEOS→SiO_2_ route introduces abundant hydroxyl groups that depress crystallinity, toughen interfaces, and scaffold char; the composite achieves σ ≈ 1.8 × 10^−4^ S·cm^−1^ at 30 °C, t^+^ ≈ 0.42, a critical current density near 1.4 mA·cm^−2^, and LFP‖Li performance of 161.2 mAh·g^−1^ at 0.5 C with about 88% retention after 400 cycles at 30 °C [107]. Taken together, these data show that dispersion quality and surface chemistry decide whether ceramic fillers act only as thermal shields or also unlock transport and interfacial benefits. A practical loading window near 5–10 wt% silicate balances barrier formation with ion transport, whereas higher contents risk over-mineralizing the matrix and raising impedance.

#### 4.5.2. Silicon-Containing Polymers

**Figure 9:**
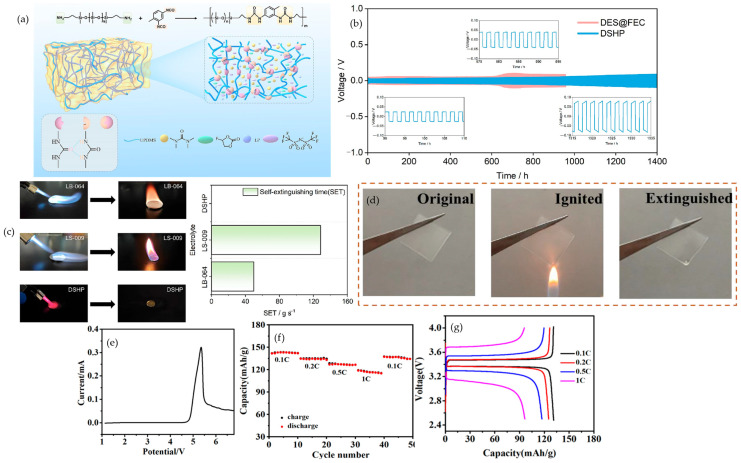
(**a**) Design and mechanistic schematic of the siloxane–deep-eutectic-solvent self-healing network. (**b**) Symmetric Li‖Li plating–stripping demonstrating long-duration interfacial stability with the siloxane gel electrolyte. (**c**) Direct-flame exposure of the siloxane gel showing non-ignition and rapid self-extinguishing. (**d**) Direct-flame test of a silicon–phosphorus copolymer membrane showing non-ignition and a dense post-burn residue. (**e**) Anodic stability by linear-sweep voltammetry. (**f**) Rate capability of LFP‖Li full cells using the silicon–phosphorus copolymer electrolyte. (**g**) Cycling performance of LFP‖Li full cells using the silicon–phosphorus copolymer electrolyte. Panels (**a**–**c**) reproduced with permission from Ref. [108] (Elsevier, 2024); panels (**d**–**g**) reproduced from Ref. [109] (MDPI, 2020).

Polymeric silicon frameworks internalize the barrier function by converting Si-O-Si backbones into SiO_2_ or SiOC under heat. The resulting glassy char blocks oxygen and stabilizes geometry, so dripping is suppressed and flammable volatiles are reduced [19,72]. Silicone-doped polyethers prepared by copolymerizing a cyclic siloxane with solvating monomers remain dimensionally stable at about 70 °C where siloxane-free controls flow, which signals effective in-matrix ceramization, as demonstrated by Chen et al. PDMS-based crosslinked electrolytes often show effectively zero self-extinguishing time during flame challenges because PDMS domains convert rapidly to silica-like residues. Quasi-solid siloxane self-healing gels with deep-eutectic solvents display the same non-combustible response under direct flame as an elastic silicone network densifies into a protective layer on heating. Figure 9a shows the siloxane–deep-eutectic-solvent network and the self-healing logic that supports barrier formation, and Figure 9c shows non-ignition with rapid self-extinguishing under a torch. The electrochemistry aligns with this safety picture rather than opposing it. Low-Tg siloxane segments raise amorphous content and, when covalently integrated with EO-rich or ionic domains, preserve Li^+^ coordination and mobility. Figure 9b shows symmetric Li‖Li plating and stripping at 0.05 mA·cm^−2^ that remains stable for about 1400 h with lower polarization than a deep-eutectic-solvent control, which confirms that ceramizing silicone domains can coexist with long-term interfacial stability [108]. Crosslinked siloxane solid polymer electrolytes (SPEs) illustrate that fire safety and performance can coexist. Kalybekkyzy et al. synthesized UV-cured PEGDA/ETPTA/a-PDMS/LiTFSI films that exhibit high thermal resilience (T10 ≈ 320 °C; T50 ≈ 380 °C) alongside a wide oxidative window to ≈5 V at 60 °C. Ionic conductivity reaches ≈1.75 × 10^−6^ S·cm^−1^ at 25 °C and ≈1.07 × 10^−4^ S·cm^−1^ at 80 °C, while Li‖Li symmetric cells cycle stably for ≈90 h at 0.2 mA·cm^−2^ without shorting, evidence of a robust SPE‖Li interface. The UV-crosslinked, amorphous network suppresses crystallinity yet preserves flexibility, enabling durable coin-cell operation [109]. Collectively, these data show that tuning segmental mobility (via PDMS) and network density can deliver polymer electrolytes with elevated thermal indicators and strong electrochemical and battery metrics for safer high-voltage Li-ion systems.

#### 4.5.3. Polyhedral Oligomeric Silsesquioxanes (POSSs)

POSS cages operate as discrete nano-ceramic nodes, as shown by Chen and co-workers. They do not contribute fuel or support gas-phase combustion; instead, the silsesquioxane core transforms into SiO_2_ micro-networks that stitch and vitrify the char while organic arms such as epoxy, acrylate, or glycidyl enforce uniform dispersion and covalent anchoring so phase separation is minimized. When an epoxy-functional POSS crosslinks a poly(ionic-liquid) ionogel, the electrolyte carries approximately 2.5 mS·cm^−1^ at room temperature with high lithium-ion mobility, and LFP‖Li cells deliver near-theoretical capacity at low rates together with useful power at elevated rates and stable cycling to hundreds of cycles. Direct-flame exposure shows a non-igniting response, and soft-pack demonstrations retain function even after mechanical damage. These features indicate that POSS supplies both mechanical reinforcement and a condensed-phase route to fire resistance without sacrificing power capability. Since effective loadings lie around 5–10 wt%, conductivity penalties remain minor; in EO-rich hosts, the cages immobilize anions near interfaces and open free volume so that modulus and toughness rise while ionic pathways remain open [110]. The overall lesson is that molecularly precise ceramic nodes can deliver the same barrier logic as particulate silica yet with tighter control over dispersion, crosslink density, and network topology.

#### 4.5.4. Synergistic Si–P Systems

Silicon and phosphorus act on complementary levers. Silicon drives ceramization and builds a rigid, low-permeability scaffold in the condensed phase, while phosphorus accelerates char formation and quenches flame radicals through PO-based species. The cooperative result is a cohesive glaze that resists back-radiation and oxygen diffusion at lower total additive content than either element alone, which explains the frequent outperformance of Si-P formulations in flammability tests and calorimetry. A PEG-based copolymer that integrates DOPO-derived phosphorus with siloxane provides a clear battery-relevant example, as demonstrated by Zeng and co-workers. Figure 9d shows direct-flame images that confirm non-ignition, rapid self-extinguishing, and a dense silica–phosphate residue. Figure 9e shows the oxidative stability window extending to about 4.8 V versus Li^+^/Li in linear sweep voltammetry. In full cells, discharge capacities after ten cycles reach approximately 142.0, 133.2, 126.6, and 115.5 mAh·g^−1^ at 0.1 C, 0.2 C, 0.5 C, and 1 C. Figure 9f shows the rate capability across these current densities, and Figure 9g shows the cycling profile with capacity recovery to about 134.5 mAh·g^−1^ when the rate returns to 0.1 C and a charge–discharge plateau gap near 0.12 V at 0.1 C, which indicates low polarization. These battery metrics sit alongside room-temperature ionic conductivity of 2.98 × 10^−5^ S·cm^−1^ and sustained cycling at 60 °C around 129 mAh·g^−1^ at 0.2 C over 100 cycles [111]. Particle-scale hybrids follow the same division of labor. Core–shell SiO_2_@AlPO_4_ fillers in PEO raise limiting oxygen index, reduce total heat release, strengthen the membrane, and can even increase conductivity, which illustrates how phosphorus makes the char while silicon builds the ceramic framework within it, as shown by Wang and colleagues [112]. A silicon–phosphorus hybrid that uses phosphazene-loaded halloysite nanotubes in PEO demonstrates the same logic at the nanotubular scale, with peak heat release reduced by a significant margin, ionic conductivity maintained within the practical 10^−5^ to 10^−3^ S·cm^−1^ window, extended electrochemical stability, and stable LFP full-cell operation. Device-level demonstrations show LEDs that remain lit even under folding, cutting, or flame exposure, which links condensed-phase glaze formation directly to abuse tolerance and practical safety, as illustrated by Zhang and co-authors [113]. Read across all four sections, a coherent picture emerges. Condensed-phase action is the cornerstone of fire safety in polymer electrolytes, yet it need not be purchased at the expense of transport or cyclability. Dispersed silica and layered silicates set the baseline by combining heat absorption, catalytic charring, and surface-anion regulation. Polymeric siloxanes internalize the barrier and, when architected as interpenetrating or block networks, preserve lithium coordination while converting to silica-like residues under heat. POSS cages refine this strategy with molecularly defined ceramic nodes. Silicon–phosphorus hybrids then combine rapid char chemistry with rigid ceramic scaffolds so that non-ignition, self-extinguishing behavior, and low heat release align with wide oxidative windows, long-lived Li‖Li stability, and robust LFP‖Li cycling.

Silicon-based approaches use porous or hydrogen-bonding silica and layered silicates to lower crystallinity and sustain room-temperature ion transport, while rigid Si frameworks strengthen mechanical and thermal stability and enhance flame behavior, and siloxane/PDMS/POSS networks add flexibility and, in some cases, self-healing. The main trade-off is that higher filler fraction and stiffer Si-based networks constrain ion-transport pathways and interfacial dynamics, tending to reduce room-temperature conductivity and rate capability. Table 6 summarizes the pros, cons, core trade-offs, and design levers.

### 4.6. Bio-Based Additives

**Figure 10:**
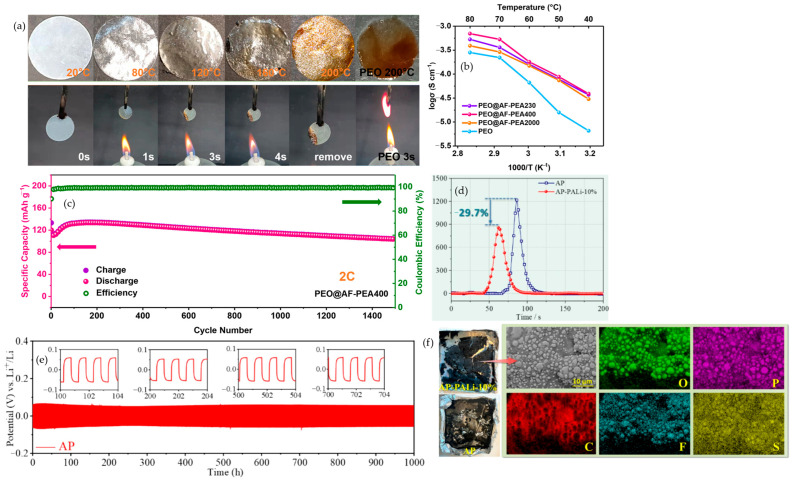
(**a**) Direct-flame images of a PEO membrane hosted in an alginate-fiber-grafted polyetheramine scaffold showing self-extinguishing and hot-shape retention. (**b**) Temperature-dependent conductivity indicating enhanced ion transport for the fiber-reinforced membrane relative to neat PEO. (**c**) LiFePO_4_‖Li full-cell cycling at elevated rate and temperature with stable capacity and high coulombic efficiency. (**d**) Cone calorimetry heat-release trace for an α-cyclodextrin polyrotaxane with immobilized lithium phytate showing a lower peak and reduced total heat. (**e**) Symmetric Li‖Li plating–stripping is stable over an extended duration with low overpotential. (**f**) Post-burn char microstructure and EDS maps revealing compact P- and O-rich domains consistent with phosphate-catalyzed dehydration and a dense condensed-phase barrier. Panels (**a**–**c**) reproduced with permission from Ref. [118] (ACS, 2022); panels (**d**–**f**) reproduced with permission from Ref. [119] (Elsevier, 2025).

Fire safety in solid polymer electrolytes is most persuasive when the protective function is encoded in the electrolyte architecture. Bio-based components enable this through coordinated roles. Carbon-rich backbones form early condensed-phase char that limits fuel release and heat feedback. Chemically tethered phosphorus remains at the dehydration front, so the char densifies where heat is highest rather than venting volatile fragments. Mineral or supramolecular motifs stiffen the barrier at elevated temperature and restrain hot flow while preserving continuous pathways for Li^+^ transport. Across recent syntheses and reviews, this architecture-first logic shows that biomass-derived elements can replace persistent additives while meeting conductivity and voltage requirements [72,114,115,116]. The shift from simple blending to deliberate architecture makes mechanisms and trade-offs explicit. A concise baseline comes from a PEO film blended with sodium alginate, reported by Y. Chen and co-workers. The limiting oxygen index rises to 28.6%, the film maintains its geometry above 120 °C, and the electrochemical window remains above 4.6 V. These outcomes indicate that condensed-phase protection can emerge without abandoning the room-temperature conductivity band typical of PEO solids, establishing a bio-enabled starting point that does not sacrifice electrochemistry [117]. Architecture then turns alginate into an active framework. Wang and colleagues graft an alginate-fiber network to a polyetheramine backbone and use it to host PEO. The composite self-extinguishes under flame and resists hot deformation, as shown in Figure 10a. Ion transport remains robust as the barrier strengthens. The conductivity–temperature trace in Figure 10b shows PEO@AF-PEA400 approaching 6.7 × 10^−4^ S·cm^−1^ at 80 °C, higher than neat PEO, which indicates preserved through-thickness percolation for Li^+^. The same architecture carries to cells. Figure 10c shows LiFePO_4_‖Li cycling at 2 C and 80 °C that remains near 103.5 mAh·g^−1^ after 1500 cycles with high coulombic efficiency and no short circuit, consistent with more uniform current distribution and better tolerance to dendrite-driven stress [118]. A complementary molecular route executes intumescence inside the matrix. A slide-ring host threads PEO with α-cyclodextrin, and immobilized lithium phytate places the acid function where dehydration proceeds. Q. Chen’s team tracks the fuel-to-barrier transition by cone calorimetry. In Figure 10d, the peak heat-release rate sits near 857 kW·m^−2^ with a 29.7% reduction relative to the reference, and total heat and smoke also decline. Electrochemical response remains serviceable. Symmetric Li cells plate and strip for roughly 1000 h at 0.1 mA·cm^−2^ and 0.05 mAh·cm^−2^ with low overpotential, as shown in Figure 10e. Post-burn structure explains the safety gain without a transport penalty. Figure 10f shows a compact residue with P- and O-rich domains and sphere-like regions, consistent with phosphate-catalyzed dehydration that locks phosphorus at the char front and builds a dense condensed-phase barrier [119]. Miniaturization retains the balance when architecture governs flame chemistry. An electrospun calcium–alginate membrane in PEO yields ionic conductivity of 3.86 × 10^−4^ S cm^−1^ at 30 °C, an electrochemical window of 5.32 V, and stable Li‖Li plating and stripping for 3000 h at 30 °C, as reported by Liu and co-workers. In LiFePO_4_‖Li cells, the same scaffold sustains 141.2 mAh·g^−1^ with 92.5% capacity retention after 300 cycles at 0.3 C and 30 °C. Calcium crosslinks stiffen the barrier, and the nanofiber network throttles heat and oxidant ingress, while through-pores maintain Li^+^ migration [120]. A renewable inorganic–organic reinforcement reaches a similar endpoint when acid functionality is built into cellulose. Aziam and co-authors show that phosphate-grafted cellulose nanofibers in PEO deliver conductivity above 10^−4^ S·cm^−1^ at 70 °C and an electrochemical stability window above 5 V versus Li/Li^+^. Flame tests and thermal analysis indicate improved fire behavior that matches acid-catalyzed dehydration and a mineral-anchored char that resists melt flow [121].

Bio-based polymer electrolytes are renewable and provide polar sites for salt dissociation, with porous or fibrillar architectures that can sustain room-temperature transport and mechanical integrity while char-forming chemistry improves fire performance. Key liabilities are hygroscopicity, modest room-temperature conductivity without mobility enhancers, strong interactions that constrain mobility, and high-voltage interfacial challenges. The central trade-off is that higher bio-content or tighter networks boost safety and stiffness but narrow ion pathways and raise interfacial losses. Table 7 presents the essentials at a glance.

### 4.7. Ionic Liquids

Ionic liquids (ILs) are salts of bulky organic cations paired with weakly coordinating anions that remain liquid near ambient conditions. Because they have negligible vapor pressure, high thermal tolerance, and wide electrochemical stability, ILs remove the flammable-vapor pathway that plagues carbonate-plasticized polymer electrolytes while still supporting ion transport when embedded in solid or quasi-solid matrices. From a safety perspective, their action is twofold. In the gas phase, ultra-low volatility starves incipient flames of fuel. In the condensed phase, ILs promote early char and stabilize inorganic–organic barrier layers that curb melt flow and dripping. These advantages persist when ILs are immobilized, either by confining them inside rigid scaffolds such as ceramics or mesoporous oxides or by polymerizing the ionic motif to create poly(ionic-liquid) solids. In both cases, the IL-rich phase remains shape-stable at elevated temperature while maintaining percolated ion pathways [122,123]. The following subsections examine these architectures and show, with quantitative data, how flame safety and electrochemical performance can be co-optimized.

#### 4.7.1. IL-Confined Composites

**Figure 11:**
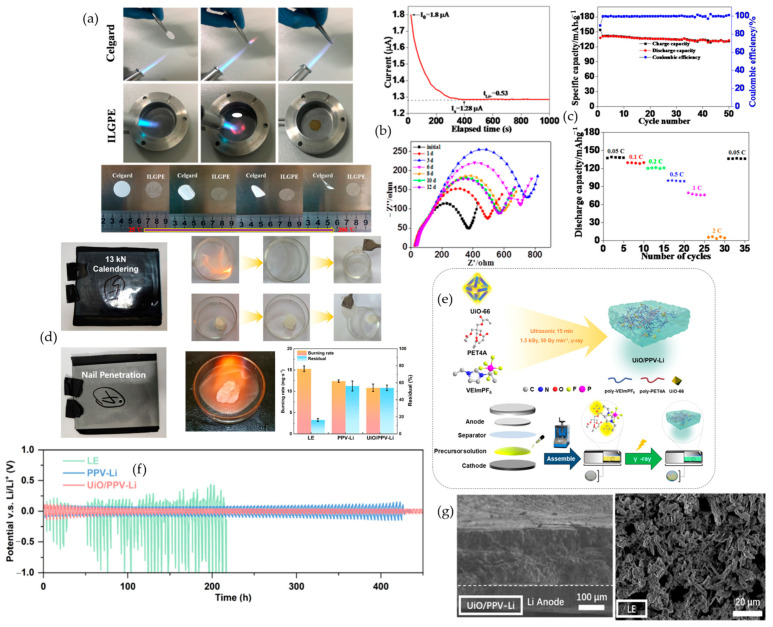
(**a**) Direct-flame non-ignition of the gel laminate. (**b**) (**Top**): Chronoamperometry profiles of the Li/ILGPE-10% LAGP/Li symmetric cell under a 10 mV step potential; (**Bottom**): Time evolution of the impedance response (EIS) of the Li/ILGPE-10% LAGP/Li cell. (**c**) (**Top**): Cycling performance of the LiFePO_4_/ILGPE-10% LAGP/Li cell; (**Bottom**): Rate-capability behavior at varied rates. (**d**) Mechanical and thermal safety of UiO/PPV-Li electrolyte and cells. (**e**) Schematic illustrating radiation-induced synthesis of UiO/PPV-Li and the assembly of a lithium-metal battery. (**f**) Voltage–time profiles of Li‖Li symmetric cells using LE, PPV-Li, and UiO/PPV-Li electrolytes at 0.5 mA·cm^−2^ over 450 h. (**g**) SEM of cycled Li‖Li symmetric cells; left—cross-sectional SEM of a Li‖UiO/PPV-Li‖Li cell; right—surface morphology after cycling with a liquid electrolyte. Panels (**a**–**c**) reproduced with permission from Ref. [125] (Elsevier, 2018); panels (**d**–**g**) reproduced with permission from Ref. [127] (Elsevier, 2025).

Confining the ionic liquid inside a rigid, tortuous host removes the vapor–fuel path for ignition and arrests flow at elevated temperature while preserving liquid-like transport and easing interfacial transfer. Kuo et al. blended an imidazolium oligomeric ionic liquid into PVDF-HFP and formed a quasi-solid membrane that is intrinsically nonflammable with a limiting oxygen index around 29, remains dimensionally stable at 150 °C with minimal change, and conducts near 2.0 mS·cm^−1^ at 30 °C and about 6.6 mS·cm^−1^ at 80 °C. The low interfacial resistance sustains high LiFePO_4_ capacity with approximately 99% coulombic efficiency over extended cycling [124]. Guo et al. generalized the same confinement logic by introducing an active NASICON scaffold. In LAGP-supported ionic-liquid gel polymer electrolytes, the oxide depresses PVDF-HFP crystallinity and supplies Li^+^ pathways, giving about 7.6 × 10^−4^ S·cm^−1^ at 25 °C for 10 wt % LAGP and supporting an oxidative limit near 4.8 V for LiFePO_4_‖Li full cells. The fire behavior appears directly in Figure 11a, which shows no sustained burning under a spray-torch for the composite. Battery output is summarized in Figure 11c, which plots the specific discharge capacity across current rates and links high-rate losses to transport limitations of gels. Transport metrics that underwrite these results are gathered in Figure 11b, which shows that the composite reaches 0.76 × 10^−3^ S·cm^−1^ at 25 °C and that the Li^+^ transference number is about 0.54 compared with about 0.46 without LAGP [125]. Rana et al. extended the safety and performance envelope with a PVDF-HFP BMIM[TFSI] LiTFSI gel prepared by controlled-temperature casting that exhibits 3.31 × 10^−3^ S·cm^−1^ at 25 °C, an anodic limit close to 5.3 V, a thermal onset around 339 °C, no sustained burning under a torch, more than 1400 h of Li‖Li stability, and LiFePO_4_ capacities of about 147.7 mAh·g^−1^ at 1 C with roughly 82% retention over 200 cycles [126]. Across these confined composites, the common outcome is no sustained ignition in flame checks, room-temperature conductivity in the 10^−3^ S cm^−1^ class, oxidative limits from about 4.8 to 5.3 V, and durable Li‖Li and Li‖cathode operation from ambient to elevated temperature.

#### 4.7.2. Poly(Ionic Liquid)

Poly(ionic liquid)s convert the ionic liquid from a plasticizer into the electrolyte matrix, which removes vapor fuel while keeping continuous ion-transport domains so flame safety and electrochemistry improve together. Xie et al. created a UiO-66–poly(ionic-liquid) radiation gel in which Zr-O clusters coordinate PF_6_^−^ and widen the oxidative window to about 5.31 V, and the crosslinked imidazolium network provides roughly 9 mS·cm^−1^ at room temperature. Device-level safety is captured in Figure 11d, which shows graphite‖LCO pouch cells that do not ignite during 13 kN calendaring and do not ignite during nail penetration. The transport mechanism is clarified in Figure 11e, which depicts a desolvation-assisted Li^+^ pathway that lowers the desolvation barrier and supports higher transference and lower interfacial polarization. Long-life lithium deposition is shown in Figure 11f, which records plating and stripping with low overpotential over hundreds of hours, and morphology is confirmed in Figure 11g, which shows smoother lithium and a more uniform interphase in SEM. The same gel delivers LiNi_0.8_Co_0.1_Mn_0.1_O_2_ capacities of about 154 mAh·g^−1^ at 1 C with strong rate performance [127]. Tang et al. confirmed the trend with a fiber-reinforced PIL inside a porous PAN scaffold that reaches about 0.32 mS·cm^−1^ at 30 °C, operates to roughly 4.8 V, supports more than 1000 h in Li‖Li symmetry, and powers LFP and NCM full cells as well as a bendable pouch device [128]. Liang et al. reported a double-network PIL ionogel that is nonflammable and maintains about 1.8 × 10^−3^ S·cm^−1^ at room temperature, an oxidative limit near 5.0 V, a Li^+^ transference number around 0.33, and LiFePO_4_ capacities near 150.5 mAh·g^−1^ for more than 200 cycles [129]. Hu et al. showed that a porous PIL and P(VDF-HFP) scaffold remains fire-resistant and sustains about 1.78 × 10^−3^ S·cm^−1^ at 25 °C with a 4.2 V window and an elevated transference number relative to Celgard [130]. Taken together, these systems indicate that fixing the ionic motif, coordinating anions with rigid hosts, tuning side chains and weakly coordinating anions, and applying light crosslinking or double-network reinforcement can raise hot-state modulus without collapsing ion percolation so flame resistance, rate capability, and cycle life advance in step.

Ionic-liquid and poly(ionic-liquid) electrolytes broaden the safety and voltage window and provide strong room-temperature transport with good wetting. IL-rich gels are intrinsically nonflammable and highly conductive but often have low lithium transference and soft matrices unless immobilized, while PIL frameworks improve lithium selectivity and shape stability but can suppress mobility when too dry or highly crosslinked. Details are consolidated in Table 8.

### 4.8. Matrix-Engineered Fire-Safe Polymer Electrolytes

Rather than treating the polymer electrolyte as a homogeneous host that depends on dissolved flame retardants, matrix-engineered designs place thermal triggers and condensed-phase barriers exactly where they are needed while preserving continuous Li^+^ pathways. By structuring the electrolyte in space with thermally responsive skins that shut down and anisotropic faces that steer wetting and heat, these architectures can suppress ignition and delay thermal runaway without sacrificing room-temperature conductivity or cycling stability. The two-representative polymer–electrolyte archetypes below emphasize experimental outcomes and report both flame metrics (UL-94, LOI, SET, pHRR) and electrochemical metrics (RT σ, tLi^+^, electrochemical stability window, and long-term cycling in Li‖Li and Li‖cathode cells).

#### 4.8.1. Sandwich Polymer Electrolytes

**Figure 12:**
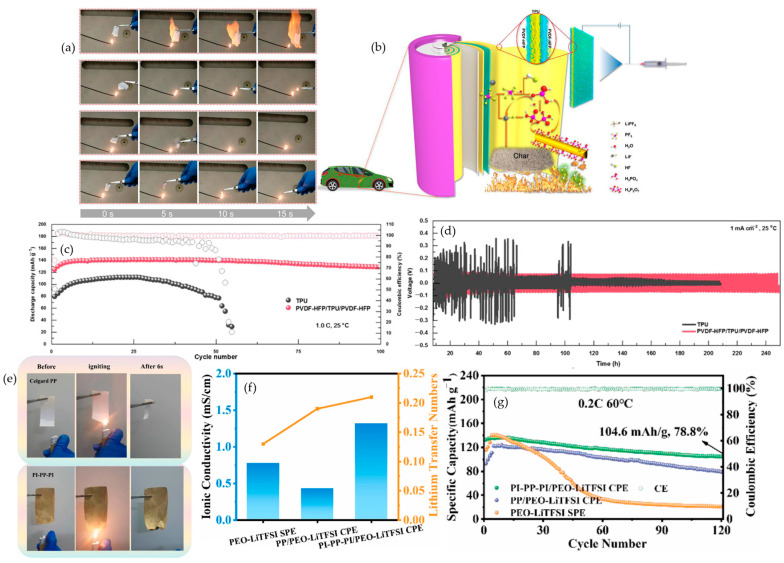
(**a**) Flammability test (top→bottom): glass fiber separator + 1 M LiPF_6_ in EC/DMC (*v*/*v*); TPU; TPU + EC/DMC (*v*/*v*); TPU + 1 M LiPF_6_ in EC/DMC (*v*/*v*). (**b**) Preparation schematic of a PVDF-HFP/TPU/PVDF-HFP nanofiber membrane and LiPF_6_-mediated flame-retardant mechanism in TPU. (**c**) Cycling at 1 C for LFP‖TPU‖Li vs. LFP‖PVDF-HFP/TPU/PVDF-HFP‖Li. (**d**) Li‖Li galvanostatic profiles with TPU and PVDF-HFP/TPU/PVDF-HFP GPE (1 mAh·cm^−2^, 1 mA·cm^−2^). (**e**) Thermal shape retention and self-extinguishing of the solid sandwich relative to PP. (**f**) Ionic-transport overview of the solid skins (conductivity and Li^+^ transference number). (**g**) Full-cell LFP‖Li cycling behavior of the solid sandwich. Panels (**a**–**d**) reproduced with permission from Ref. [132] (Elsevier, 2022); panels (**e**–**g**) reproduced with permission from Ref. [133] (Elsevier, 2025).

Sandwich architecture builds thermally responsive skins around an ion-rich core. Under abuse, the outer layers densify, and condensed-phase reactions erect a transient barrier that raises impedance, throttles heat and mass transfer, and suppresses ignition; under routine operation, the interior maintains continuous Li^+^ pathways and low polarization. Xie et al. [131] first established the self-shutdown principle in a PVDF-HFP gel with a poly-pentaerythritol tetraacrylate (PETEA) core. Near 80 °C, the membrane shows a sharp resistance rise that gates Li^+^ flux before runaway can cascade, yet LFP‖Li still delivers about 135 mAh·g^−1^ at 1 C with more than 95% capacity retention after 200 cycles, and symmetric Li‖Li cells sustain smooth plating for over 1000 h. The conductivity collapse is nearly seven orders of magnitude, reaching on the order of 10^−8^ S·cm^−1^ at 80 °C, and dye-tracing confirms that through-diffusion is arrested after heating. This result sets the causal template: a heat-triggered barrier forms where transport is most sensitive, interrupting thermal feedback without compromising room-temperature conduction and cycling. Building on that template, Han et al. [132] translate the concept to carbonate gels by laminating PVDF-HFP/thermoplastic polyurethane (TPU)/PVDF-HFP, and the material responds precisely as the mechanism predicts. Direct-flame sequences in Figure 12a show non-ignition at the membrane scale. Figure 12b traces LiPF_6_ thermolysis to PF_5_ and subsequent hydrolysis to polyphosphoric species that passivate the hot surface, consolidate a phosphate-rich char, and dilute fuel in the flame zone, which is the condensed-phase analogue of Xie’s impedance gate. The electrochemical outcome is consistent. Figure 12c, d shows lower interfacial impedance than Celgard, roughly 250 h of Li‖Li cycling at 1 mA·cm^−2^ with 1 mAh·cm^−2^, and improved LFP‖Li polarization and capacity compared with a TPU-only gel, tying the flame-blocking skin directly to cleaner interfaces and steadier lithium deposition. Cone calorimetry and post-burn residues indicate a higher char yield on the order of tens of percentage points and a reduction in total heat release, and the fiber laminate remains mechanically intact at test temperatures relevant to abuse. He et al. [133] then extend the same safety logic to an all-solid laminate by placing polyimide skins on polypropylene and infusing PEO-LiTFSI, converting the chemical shutdown and char-forming ideas into a mechanically robust thermal shield. Figure 12e shows shape retention at elevated temperature and self-extinguishing relative to PP, with the composite preserving geometry at 150–180 °C while PP alone softens and shrinks. Transport remains in a useful regime. Figure 12f reports an ionic conductivity around 1.32 mS cm^−1^ at 60 °C and a Li^+^ transference number near 0.21, indicating that the safety layer does not choke ion flow. Device-level behavior aligns with these materials metrics. Figure 12g shows high-coulombic-efficiency LFP‖Li cycling over 120–150 cycles, consistent with symmetric Li cells resisting dendrites for about 1600 h at 0.1 mA·cm^−2^, and the laminate maintains low polarization under the same current density.

In brief, sandwich architectures tailor the electrolyte to pair safety with interfacial control. Sandwich designs insert a reinforcing or nonflammable core that lifts mechanical stability, self-shutdown behavior, and dendrite tolerance, but added interfaces and thickness can raise polarization if porosity or bonding is mistuned. The key points are summarized in Table 9.

#### 4.8.2. Janus/Gradient Gel Polymer Electrolytes

**Figure 13:**
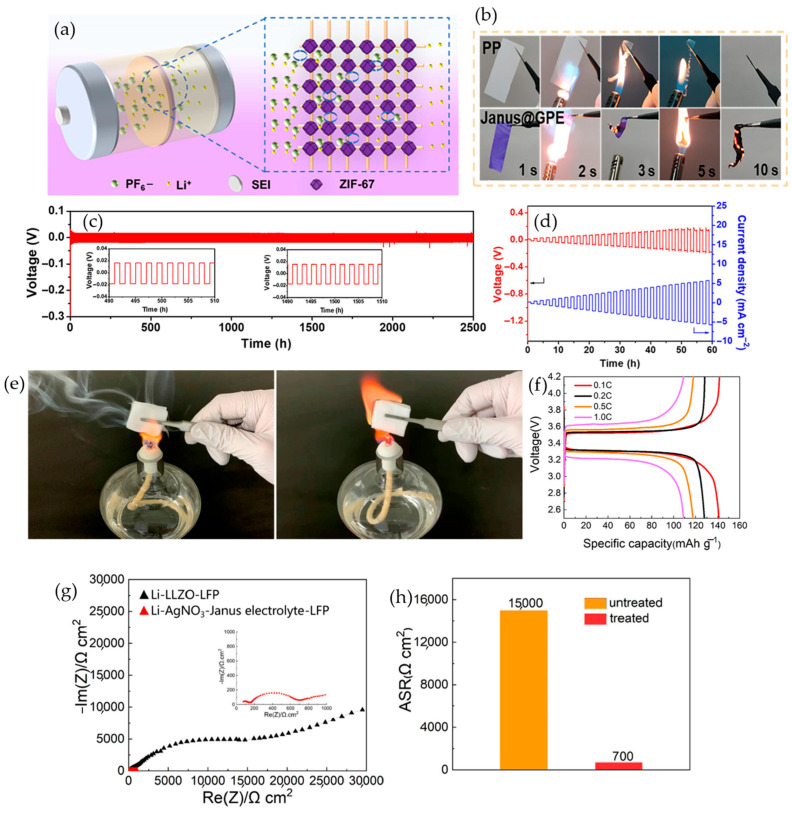
(**a**) Mechanistic schematic of a Janus gel polymer electrolyte showing Li-facing flux regulation and cathode-facing wetting/heat spreading. (**b**) Direct-flame response of the Janus gel showing self-extinguishing behavior. (**c**) Symmetric Li‖Li plating–stripping stability of the Janus gel. (**d**) Critical-current-density evaluation comparing Janus gel and control electrolyte. (**e**) Torch test of an electrochemically matched Janus solid electrolyte showing nonflammability. (**f**) Charge and discharge profiles at different rates of Janus solid electrolytes. (**g**) EIS spectra of Li|AgNO3-Janus electrolyte|LiFePO4 and Li|LLZO|LiFePO4 cells. (**h**) Interfacial-resistance comparison between the two cells. Panels (**a**–**d**) reproduced from Ref. [134] (Wiley, 2024); panels (**e**–**h**) reproduced with permission from Ref. [135] (ACS, 2021).

From a fire-safety standpoint, the essential value of Janus GPEs is the asymmetric layout that assigns functions to the faces where hazards originate. The Li-facing layer moderates Li^+^ flux to suppress local current spikes and dendrite-seeded hot spots, while the cathode-facing layer improves wetting and spreads heat to manage incipient micro-shorts, as illustrated in Figure 13a. Beyond the schematic, the gel exhibits room-temperature ionic conductivity around 1.0 × 10^−3^ S·cm^−1^, a transport activation energy near 0.49 eV, and a Li^+^ transference number close to 0.57. These figures limit interfacial polarization and flatten salt-concentration gradients during plating and stripping, and the stability window approaches 4.8 V vs. Li^+^/Li. Consistent with this mechanism, the membrane self-extinguishes in about 3 s while maintaining gel-regime transport, as documented in Figure 13b [134]. Durability under load follows from the transport: Li‖Li cycling persists for roughly 2500 h at 1 mA cm^−2^, and the critical current density rises from about 0.6 mA·cm^−2^ with a conventional gel to about 2.7 mA cm^−2^ with Janus@GPE, as shown in Figure 13c,d. The same face-selective logic is carried into an electrochemically matched solid Janus electrolyte that is intrinsically nonflammable. Direct-flame testing records no ignition and no sustained burning, as seen in Figure 13e, and thermogravimetric analysis places thermal stability near 330 °C. Interface engineering connects this safety to performance. On the cathode side, Nyquist spectra show a large impedance drop with the matched Janus configuration; the area-specific resistance falls from about 15 000 Ω·cm^2^ to roughly 700 Ω·cm^2^, as summarized in Figure 13g,h. On the anode side, a simple AgNO_3_ pretreatment of LLZO forms a thin Li-Ag interlayer that lowers interfacial resistance to about 35 Ω·cm^2^ and boosts the critical current density by roughly a factor of seventeen, enabling stable symmetric operation at 0.2 mA cm^−2^ for about 300 h without rising polarization. Under rate stress, full cells at room temperature deliver about 115 mAh·g^−1^ at 1 C for one hundred cycles with nearly unity coulombic efficiency and retain about forty-five percent of nominal LFP capacity at 2 C, as presented in Figure 13f [135]. Independent reports, as shown by Xiang et al. using a nanofiber Janus CPE (PEO/poly-*m*-phenyleneisophthalamide (PMIA) × oxygen-vacancy-enriched yttrium oxide (Y_2_O_3_)-doped zirconium dioxide (YSZ)), apply the same face-selective architecture: oxygen-vacancy-rich YSZ on the Li-facing side forms percolated ceramic pathways and binds anions to release more Li^+^, while the opposite PMIA side provides robust mechanical support and heat tolerance. At 50 °C, these CPEs reach an ionic transference number of 0.50 with σ ≈ 1.6 × 10^−4^ S cm^−1^, suppress dendrites for > 2500 h in Li‖Li, sustain 5000 LFP cycles at 1 C, and keep pouch cells operating under thermal abuse up to 145 °C without deformation [136]. Placed side by side, these results read as a continuous argument rather than isolated case studies. The gel system establishes that directing ion transport and heat management to the correct faces can deliver rapid self-extinguishing behavior within three seconds while expanding the dendrite-free operating window from about 0.6 to about 2.7 mA cm^−2^. The solid system then shows how the same architectural principle, when electrochemically matched and coupled to low interfacial resistance, suppresses local heating in routine service and removes the fuel path during abuse, yielding non-ignition under a torch and stable room-temperature power delivery. In sequence, Figure 13 moves from architecture and mechanism to flame response and then to interfacial metrics and full-cell outcomes, and the combined evidence supports a single conclusion: Janus design enables intrinsic fire safety by controlling Li^+^ flux and stabilizing interfaces during operation while ensuring a fast condensed-phase response and limited gas-phase fuel under fault, so safety gains are obtained without trading away electrochemistry.

In summary, Janus architectures tailor the electrolyte to pair safety with interfacial control. Janus designs program an anode-facing lithiophilic side and a cathode-facing oxidation-resistant side to stabilize both interfaces, yet they demand correct orientation and balanced gradients to avoid transport imbalance. The net trade-off is higher abuse tolerance and interface specificity versus through-plane conductivity and process complexity. The key points are summarized in Table 10.

In brief, flame-safety strategies for polymer electrolytes span chemical additives, inorganic frameworks and fillers, bio-derived hosts, IL/PIL ionogels, and architected designs such as sandwich and Janus. Each route balances suppression mechanisms with ion-transport retention, targets for voltage/temperature/cycling safety, and processing constraints. Table 11 presents a decision matrix mapping mechanisms, practical loadings, performance and safety targets, processing priorities, and the effectiveness of the mentioned strategies.

## 5. Conclusions and Outlook

This section distills how chemistry and architecture can be co-designed to deliver intrinsic fire safety without sacrificing ion transport and then translates those principles into manufacturable, sustainable solutions that hold up at the device level.

### 5.1. Summary of Mechanistic Design Principles and Architectures

Intrinsic safety arises when the electrolyte forms an ion-permeable condensed-phase barrier that damps heat and mass feedback while preserving clean Li^+^ pathways. Phosphorus working with nitrogen or silicon tightens and mineralizes the char, yielding thin benign residues that tolerate higher voltage and avoid halogen corrosion risks. Inorganic fillers add endothermic cooling and mineral skins and, when organized as percolated or lightly aligned networks, open Li^+^ pathways at modest loading and stiffen regions near lithium. Layered double hydroxides are a robust halogen-free option whose tunable interlayers help keep loading low without sacrificing flame performance or transport. Ionic liquids are intrinsically nonflammable, yet they should be immobilized within poly(ionic-liquid) or ionogel frameworks, with oxidation-tolerant matrices and anions for high-voltage or oxidizing environments. MOF or COF scaffolds can immobilize salts or ionic liquids and tune pore polarity and crystallinity, which helps decouple safety from conductivity; this host strategy is also evident in sulfur-facing separators where selective sorption and size screening are needed. Bio-based motifs such as phytic acid, chitosan, and lignin provide sustainable binding and early cohesive char that leaves ion-permeable residues when molecular-weight and ash windows are controlled. Function localizes effectively in layered morphologies, where sandwich membranes place flame-retardant skins around a conductive core and Janus membranes dedicate a flame-shielding face and an ion-conducting face to minimize transport penalties. Taken together, these chemistries and architectures supply a coherent toolkit for building safer polymer electrolytes while maintaining ion transport. Turning this toolkit into working membranes highlights two practical tensions and how to resolve them. Barriers that stop flames can choke diffusion if thickness, porosity, and crosslinking are not tuned for conductivity. Protective residues can foul interphases at high voltage if the chemistry is not selected for cleanliness. To compare materials fairly and manage these trade-offs, link safety to transport by reporting limiting oxygen index with specimen thickness and mass-normalized self-extinguishing time together with ionic conductivity and Li^+^ transference. Framed this way, the mechanistic levers above map directly onto roll-to-roll process rules and electrode-compatibility choices in the following sections.

### 5.2. From Blueprint to Practice: Cost, Scalability, Sustainability, and Next-Generation Compatibility

Deployable Li-ion polymer electrolytes begin with manufacturability and cost. Concentrate function at interfaces and outer skins. Build percolated or shear-induced inorganic networks using roll-to-roll slot-die or curtain coating [137]. Minimize ionic-liquid and MOF or COF fractions while keeping the condensed-phase barrier intact. Since drying and solvent recovery dominate the energy and carbon of wet coating, use continuous drying with closed-loop recovery [138]. When gradients or skins are needed, form them by controlled evaporation in dual-slot or sequential passes already demonstrated at the pilot scale [139]. Green-by-design choices reinforce this path: favor halogen-free, low-ash P-N-Si packages, use abundant mineral fillers such as layered double hydroxides, adopt bio-based binders or graftable motifs where feasible, and push toward high-solids or low-solvent coating tuned for efficient recovery. Prioritize approaches by scale-up readiness while preserving the mechanistic levers. High-readiness routes include inorganic fillers arranged into percolated or lightly aligned networks, which fit masterbatch dispersion, inline high-shear, and mild calendering, and deliver flame resistance with preserved Li^+^ transport at low loading. Halogen-free P-N-Si packages are likewise ready because they work at low dosage, leave thin benign residues, and integrate with existing mixing and coating workflows. Layered architectures created by controlled evaporation are favorable since sandwich or Janus skins can be laid down inline on tools already used for electrodes and separators. Medium-readiness options include pre-ceramic silicon additives that form conformal silica under heat, bio-based binders and flame-retardant motifs that provide early cohesive char, and immobilized ionic liquids in PIL or ionogel frameworks that retain nonflammability while curbing migration. These scale when moisture is controlled, molecular-weight windows are respected, ionogel crosslink density is tuned, and inorganic networks are used to reduce loading and viscosity. Harder-to-scale routes today include MOF or COF hosts due to material cost, synthesis and solvent management, and slurry rheology [140], while halogenated packages face corrosion and regulatory headwinds and are best reserved as laboratory benchmarks. Compatibility then narrows choices for Li-ion electrodes. For NMC, NCA, LCO, and LFP cathodes operated at 4.2 to 4.5 V, favor halogen-free P-N-Si residues and oxidation-tolerant IL or PIL matrices and avoid acidic or corrosive species that destabilize the interphase or current collectors [141]. For graphite anodes, immobilize ionic liquids and limit free-solvent activity to suppress co-intercalation; verify SEI compatibility of any condensed-phase residues [142]. Where lithium metal is used in research formats, employ higher-modulus near-anode skins from percolated or lightly aligned fillers and thin in situ interphases from pre-ceramic silicon additives with tight moisture control [143]. Two brief contrasts clarify design limits. In Li-S cells, MOF or COF pores can host polysulfides, and soft ion-permeable lithium-side skins maintain contact, which shows that any flame-retardant residue must remain thin and non-blocking [144]. In Li-O_2_ systems, the need for controlled oxygen flux and radical resistance favors ceramic-rich outer skins coupled with oxidation-tolerant ionogels or ionic liquids, which in turn support oxidation-robust matrix choices for high-voltage Li-ion [145]. Industrialization demands roll-to-roll coatability for useful solids with stable slurry rheology, clean dispersion, and tight thickness control. Cost and supply-chain robustness favor low-ash halogen-free packages and abundant mineral fillers, backed by clear bills of materials and vendor specifications. Environmental performance requires closed-loop solvent recovery, low drying energy, and explicit targets for embodied carbon per membrane area. Reliability and comparability hinge on a compact validation pack that reports ionic conductivity, Li^+^ transference, limiting oxygen index with specimen thickness, mass-normalized self-extinguishing time, and pouch-cell thermal and mechanical abuse at matched areal loadings and thickness budgets. Track line KPIs such as web speed, solids-loading windows, slurry stability and viscosity, yield, and defect density with inline or at-line metrology. Integration with Li-ion stacks calls for thin benign residues at operating voltage, SEI-compatible anode interfaces, and documented quality control and end-of-life pathways. Taken together, these process rules translate the mechanistic toolkit into membranes that are safer by design while preserving transport and manufacturability [137].

### 5.3. Green Outlook and Research Priorities

To advance flame-retardant polymer electrolytes sustainably, researchers should embed green chemistry from the start by using bio-derived monomers and non-toxic flame retardants to lower environmental burden. Priority goes to renewable or otherwise benign materials by developing polymers from biomass or recycled feedstocks, replacing halogenated additives with safer options, and designing electrolytes for end-of-life recovery and recycling. For conductive nanofillers, especially MXenes, a key future direction is fluoride-free synthesis (e.g., Lewis-acidic molten-salt routes, electrochemical chloride media, or alkali-assisted hydrothermal) to eliminate HF and fluoride effluents; leveraging -O/-OH/-Cl-terminated or phosphate/siloxane-grafted MXenes can couple heat dissipation, char promotion, and stable ionic pathways while improving process safety and recyclability. Progress should be tracked with quantitative sustainability metrics, including life-cycle assessment for carbon, water, and toxicity, together with standardized safety–performance trade-off metrics such as flammability tests and UL94 ratings. Scale-ready practice favors processes that minimize solvents and waste, employ solvent-free or low-solvent curing where possible, and improve energy efficiency in manufacturing. Engagement with manufacturers, recyclers, and regulators helps align new electrolytes with safety standards and recycling infrastructure. In conclusion, these strategies support flame safety without sacrificing environmental or operational viability, enabling designs that are safe, high-performing, and more sustainable as they move from laboratory concepts to production.

## Figures and Tables

**Table 1:** Inorganic fillers for polymer electrolytes: advantages, disadvantages, trade-offs, and design levers.

System	Advantages	Disadvantages	Trade-Offs	Design Levers	Refs.
Mineral fillers	Improves fire resistance and dimensional stability. Reduces solvent leakage. Strengthens mechanical integrity. Functional or mesoporous surfaces stabilize interfaces and preserve RT conductivity.	High loading increases tortuosity and lowers uptake. Moisture and dispersion issues raise interfacial resistance. Dense networks can depress initial capacity.	More filler increases safety and stiffness. If porosity and wetting are not maintained, room-temperature transport declines.	Use moderate loading and prefer mesoporous or functional fillers. Employ porous or electrospun hosts; tune particle size and surface chemistry. Consider hybrid mixes and mild plasticization in quasi-solids.	[56,57,58,59]
NASICON	Provides intrinsic Li-ion pathways. Lowers polymer crystallinity. Improves thermal stability and reduces shrinkage. Surface-treated particles and mesoporous partners lower interfacial resistance and improve Li compatibility.	Excess ceramic increases tortuosity and decreases uptake. Over-rigid networks reduce initial capacity. Moisture and aggregation raise interfacial resistance. Unmodified NASICON may react at Li contact.	More ceramic improves safety and dimensional stability. Without engineered porosity and interfaces, room-temperature transport declines, and an optimum loading is required.	Target moderate content and use porous or electrospun hosts. Apply surface functionalization or coatings; co-fill with mesoporous inert phases. Verify performance at RT and at elevated temperature.	[60,61,62,63,64]
MXene	Reduces polymer crystallinity and improves RT conductivity. O/OH-rich terminations promote salt dissociation and interfacial stability. Interlayers homogenize current and moderate Li nucleation.	Overloading causes electronic percolation and side reactions. Poor dispersion or oxidation increases interfacial resistance and brittleness. Dense networks reduce RT transport. O2/H2O sensitivity reduces durability.	More MXene and denser networks improve interfacial control and safety. If pathways narrow or electronic leakage occurs, room-temperature rate and efficiency decline.	Keep loading below the percolation threshold. Engineer surfaces with oxygen-rich terminations or polymer grafts. Use porous or aligned hosts; combine with inert or mesoporous fillers. Manage oxygen and moisture, and add anti-oxidation steps.	[65,66,67,69]

**Table 2:** MOFs in polymer electrolytes: advantages, disadvantages, trade-offs, and design levers.

System	Advantages	Disadvantages	Trade-Offs	Design Levers	Refs.
MOF	High surface area and ordered pores support ion transport and solvent retention. Tunable chemistry enables anion immobilization and higher Li^+^ selectivity. Rigid, porous scaffolds encapsulate flame retardants and improve dimensional stability.	Excess loading raises tortuosity and lowers uptake, weakening RT transport. Moisture/chemical sensitivity can raise interfacial resistance and aging. Pores may become blocked; open metal sites can catalyze side reactions.	More framework or pore filling strengthens safety and mechanics, while RT transport holds only if channels stay open and well-wetted. Finer, well-dispersed crystals smooth interfaces, but very small powders increase boundary resistance and cost.	Select water-stable and thermally stable MOFs (e.g., ZIF-8, UiO-66); tune pore size and functionality for Li^+^. Use nanoscale crystals or porous 3D skeletons and ensure thorough pore wetting. Anchor/encapsulate flame retardants; add surface grafts/coatings; combine with inert or MXene partners at moderate loading.	[71,74,75,76,77]

**Table 3:** COFs in polymer electrolytes: advantages, disadvantages, trade-offs, and design levers.

System	Advantages	Disadvantages	Trade-Offs	Design Levers	Refs.
COF	Lightweight ordered channels guide selective Li^+^ transport and raise transference number. Char-forming, thermally stable backbones support flame performance. Composite membranes with COFs can improve toughness and dimensional stability.	Modest intrinsic conductivity without tailored ionic groups. Overloading or high crystallinity limits polymer mobility. Pores can collapse or become blocked; interfaces may require modification.	Higher crystallinity improves safety and stiffness but reduces RT transport without open, wetted channels. Stronger pore-wall functionalization raises selectivity and t+ but can lower oxidative stability and increase cost.	Choose chemically robust 2D/3D COFs (e.g., β-ketoenamine, imine, triazine) with high thermal stability. Align or template channels and preserve porosity; functionalize pore walls for Li^+^ affinity. Grow in situ or use thin interlayers; blend with inert or MXene fillers at moderate loading.	[71,78,79,80,81,82]

**Table 4:** Phosphorous-based additives: advantages, disadvantages, trade-offs, and design levers.

System	Advantages	Disadvantages	Trade-Offs	Design Levers	Refs.
Phosphorous-based additives	Condensed-phase char and acid catalysis improve fire behavior. PO· gas-phase quenching adds suppression. Immobilized or in situ-formed species raise thermal and mechanical robustness.	Overloading reduces RT transport and raises interfacial impedance. Some chemistries are moisture- or oxidation-sensitive and may migrate if untethered. Possible high-voltage side reactions.	More P or stronger crosslinking raises fire safety and stiffness but cuts Li^+^ mobility and interfacial kinetics unless channels and wetting are maintained. Free or moisture-sensitive P can migrate or degrade. Immobilize it and keep loading moderate.	Match P chemistry to the voltage window and keep loading moderate. Prefer tethered or in situ immobilized species; use microcapsules judiciously. Passivate Li and collectors, control moisture, and co-design with low-fraction inorganic fillers.	[83,84,85,86,87,88,89,90,91,92,93,94]

**Table 5:** Halogen additives: advantages, disadvantages, trade-offs, and design levers.

System	Advantages	Disadvantages	Trade-Offs	Design Levers	Refs.
Fluorine-based additives	Strong flame resistance with optimized F content. LiF-rich interphases can stabilize Li and high-V cathodes. In situ gelation or crosslinking improves dimensional stability.	HF and COF2 on abuse plus per- and polyfluoroalkyl substance (PFAS) persistence concerns. Over-fluorination stiffens the matrix and reduces RT wetting and transport. Higher crosslink density raises impedance and processing burden.	A higher F fraction improves suppression and high-voltage stability, but stiffens the matrix and raises impedance at room temperature. Greater crosslink density stabilizes geometry, but penalizes ionic transport and processability.	Use a moderate F fraction with in situ gelation. Co-plasticize and tune crosslink density. Engineer CEI/SEI to preserve wetting and rate.	[97,98,99,100]
Bromine-based additives	Very strong gas-phase radical quenching for fast flame suppression. LiBr routes can form protective, organic-rich CEI at high voltage.	Corrosive HBr and brominated fumes on abuse with higher smoke toxicity. Regulatory pressure on organobromines and risk of metal corrosion if untethered. Transport penalties at high loading.	More Br speeds gas-phase quenching but elevates corrosion risks and hurts transport at high loading. Mobile Br species act quickly, but can migrate and age, while tethered Br is more stable but less responsive.	Tether brominated units or use LiBr at controlled levels with passivation and acid scavengers. Limit loading and pair with robust hosts or ionogels to retain mobility. Manage interphase chemistry to balance suppression and transport.	[101,102,103]

**Table 6:** Silicon fillers and networks: advantages, disadvantages, trade-offs, and design levers.

System	Advantages	Disadvantages	Trade-Offs	Design Levers	Refs.
Silicon-based additive	Porous or H-bonding surfaces lower crystallinity and support RT transport. Surface functionality and charge can aid anion management and interface stability. Rigid Si frameworks improve mechanical and thermal stability and flame behavior. Siloxane/PDMS/POSS add flexibility and potential self-healing; Si–P hybrids show char synergy.	High loading increases tortuosity and lowers uptake. Aggregation and surface traps raise interfacial losses. Some siloxanes have limited voltage windows. Possible residual volatiles and crosslinking complexity.	More framework strengthens safety and stiffness, while RT conductivity drops and interfacial impedance rises if channels are not kept open and wetted. Stronger anion binding lifts Li^+^ transference, while overall ion transport can slow.	Use moderate loading and apply surface functionalization or coatings. Control dispersion quality (e.g., ultrasonication, high shear). Adjust crosslink density and plasticization; choose siloxane backbones with adequate voltage stability. Pair with phosphorus co-FRs to reach target safety at lower total fraction.	[104,105,106,107,108,109,110,111,112,113]

**Table 7:** Bio-based electrolytes: advantages, disadvantages, trade-offs, and design levers.

System	Advantages	Disadvantages	Trade-Offs	Design Levers	Refs.
Bio-based	Renewable origin, greener profile. Polar sites enable salt dissociation and Li^+^ selectivity. Porous/fibrillar networks support RT transport and mechanical integrity. Char-forming tendency improves flammability behavior.	Hygroscopic and moisture-sensitive; leads to higher aging risk. RT conductivity is modest without mobility enhancers. Strong interactions or high crystallinity limit segmental motion. High-voltage interfaces are challenging.	Higher bio-content or tighter networks enhance safety/interphase stability but constrict pathways and slow RT kinetics. Softer matrices maintain contact but sacrifice high-T dimensional stability.	Keep loading moderate; compatibilize or functionalize with the host. Blend with PVDF-HFP or similar; add benign plasticizers; control porosity to keep channels open. Add small inorganic co-additives to share flame suppression without sacrificing transport.	[114,115,116,117,118,119,120,121]

**Table 8:** IL/PIL-based polymer electrolytes: advantages, disadvantages, trade-offs, and design levers.

System	Advantages	Disadvantages	Trade-Offs	Design Levers	Refs.
Il-based	Low volatility and intrinsically nonflammable. Wide electrochemical window. High RT conductivity with good wetting. In situ gelation gives uniform interfaces; solid supports add shape stability.	Li^+^ transference can be low unless ions are immobilized. Higher viscosity and processing complexity. Possible Al corrosion at high potentials depending on anion. ∙	More IL improves conductivity and flame safety, while softening the matrix and lowering Li^+^ selectivity. More host/scaffold improves shape and safety but narrows pathways and reduces conductivity.	Choose IL cation/anion for stability and window; optimize IL fraction. Use in situ gelation with PVDF-HFP or similar hosts. Add MOF/NASICON scaffolds and engineer CEI/SEI for clean interfaces.	[122,123,124,125,126]
PIL-based	Immobilized anions can raise Li^+^ transference. Nonflammable options with wide window. Good shape stability and flexibility. Double-network or fiber-reinforced designs reach high conductivity.	Conductivity falls if too dry or over-crosslinked. Needs IL or plasticizer for mobility. Added processing complexity; interfaces can be resistive without treatment.	More PIL and crosslinking improve safety and stiffness but reduce segmental motion and conductivity. Adding IL restores mobility but reduces Li^+^ selectivity and softens the matrix.	Tune PIL chemistry/crosslinking; optimize IL content. Use double networks; reinforce with fibers/ceramics; consider MOFs. Add surface functionalization or in situ polymerization to cut interfacial loss.	[122,127,128,129,130]

**Table 9:** Sandwich-structured polymer electrolytes: advantages, disadvantages, trade-offs, and design levers.

System	Advantages	Disadvantages	Trade-Off	Design Levers	Refs.
Sandwich-structured	Central mid-layer increases mechanical strength and dimensional stability. Shutdown or nonflammable core improves abuse and puncture tolerance. Compatible with in situ gelation for strong interlayer adhesion.	Extra interfaces can raise interfacial resistance if mismatched. Lamination/alignment adds process complexity. Added thickness lowers energy density; risk of delamination or solvent migration during cycling.	Thicker, stiffer cores boost safety, while RT conductivity and energy density drop if porosity/wetting are not preserved. Stronger interlayer bonding stabilizes cycling, while reworkability and wetting windows narrow.	Use robust mid-layers (e.g., nanofiber/TPU) with tuned porosity and thickness. Activate surfaces or use primers; lock layers by in situ gelation to cut contact resistance. Match electrochemical stability to both electrodes; control pressure and orientation during assembly.	[131,132,133]

**Table 10:** Janus-structured polymer electrolytes: advantages, disadvantages, trade-offs, and design levers.

System	Advantages	Disadvantages	Trade-Off	Design Levers	Refs.
Janus-structured	Asymmetric faces tailor each interface; lithiophilic anode face promotes uniform Li deposition. Oxidation-resistant cathode face tolerates high voltage. Nonflammable formulations lower hazard and reduce polarization.	Orientation-sensitive during assembly; steep gradients can unbalance transport. Higher synthesis and compositional control burden. Face mismatch may trigger interfacial side reactions.	Strong anode-face functionality stabilizes plating, while bulk transport can suffer if pathways on the cathode face become too rigid or fluorophilic. Robust cathode-face protection widens voltage tolerance, while added impedance and face imbalance can limit rate.	Program distinct chemistries per face; grade porosity and crosslink density through thickness. Perform in situ polymerization on each electrode for adhesion and clean interfaces. Keep total thickness low to maintain conductivity; use nonflammable, wettable hosts.	[134,135,136]

**Table 11:** Decision matrix for flame-safety strategies in polymer electrolytes.

System	Primary Mechanisms	Practical Loading	Targets	Processing Priorities	Effectiveness
Phosphorus-based	Char formation, acid catalysis, PO radical quench, P-rich interphase	Low–moderate	Self-extinguished, stable cycling	Tether or crosslink, in situ delivery, moisture control, interface passivation	High: Self-extinguishes at low dose Char and radical quench stabilize under heat Caveat: Moisture and high voltage unless tethered. Scalable with polymerizable P
Fluorine-based	Gas-phase quench, intrinsically nonflammable gels, wider oxidative window	Low–moderate	High-voltage, abuse tolerance, flexible formats	In situ gelation, crosslink tuning, CEI control, EHS management	Moderate–High: Gels rarely ignite Low volatility and radical quench reduce flame spread Caveat: HF and EHS risk and higher viscosity. Feasible with controls
Bromine- based	Strong gas-phase quench, bromide-enabled CEI	Low	Maximum suppression under abuse	Tethering, limited dose, passivation, gas handling	Moderate–High: Very potent at tiny dose Rapid chain break in flame zone Caveat: toxic and corrosive byproducts and regulation. Weak fit at scale
Mineral fillers	Heat sink and barrier, crystallinity reduction, reinforcement	Moderate	Self-extinguish trend, dimensional stability	Surface functionalization, good dispersion	Moderate: Slows ignition and spread Thermal absorption and barrier to heat and mass Caveat: higher loading can reduce conductivity and ductility. Very scalable and low cost
NASICON	Fast Li pathways, thermal stability, barrier	Moderate	Safety with transport retention, dendrite moderation	Surface treatment, porous hosts, dual fillers	Moderate: Removes fuel while keeping Li paths Stable ceramic network in polymer Caveat: Brittleness and interface resistance.
MXene	Crystallinity control, interfacial stabilization, guided Li nucleation	Low	Plating uniformity with safety kept	Surface terminations, oxidation control, hybrids	High: Strong flame resistance at about 1–2 wt% Heat spreading and char promotion at low dose Caveat: Oxidation sensitivity and electronic percolation if overdosed.
MOF	Ordered pores, anion management, FR hosting	Low–moderate	Selective transport with FR gain	Nanoscale MOFs, encapsulation, grafting	Moderate–High: Porous host aids quench and char Tunable chemistry at low loading Caveat: Moisture sensitivity and cost and dispersion control.
COF	Ordered channels, N-rich sites, thermal stability	Low–moderate	Thermal safety with selectivity, channel alignment, wall functionalization	Gentle compatibilization, morphology control, dry handling	High: Intrinsic flame resistance with maintained transport Stable covalent lattice and high char yield Caveat: Synthesis and alignment challenges.
Silicon-based	Porous or H-bond surfaces, rigid Si barrier, Si–P synergy	Moderate	Safety and stiffness with RT transport	Surface coating, dispersion, network tuning	Moderate–High: Silica skin and Si–P char raise LOI and stability Halogen-free barrier and char formation Caveat: Higher loading increases tortuosity. Some siloxanes limit voltage. Good manufacturability
Bio-based	Polar sites, fibrillar or porous networks, inherent char	Moderate	Safer cycling, greener profile	Gentle compatibilization, morphology control, dry handling	High: Intrinsically nonflammable and often self-quenching Replaces flammable carbonates and wets pores well Caveat: Low Li^+^ transference and higher viscosity, leakage risk, and cost or EHS limits
IL-based	Nonflammable, wide window, high RT conductivity, good wetting	Optimized high IL	High-voltage, abuse tolerance	In situ gelation, scaffold support, CEI/SEI design	Moderate–High: Solid and nonflammable Higher Li^+^ selectivity than IL-rich gels Caveat: Rigidity reduces RT conductivity unless plasticized or blended
PIL-based	Immobilized anions, shape-stable, low flammability	Moderate PIL	Safety with higher Li^+^ selectivity	Crosslink tuning, double networks, fiber or ceramic reinforcement	Moderate: Intumescent char and scaffolded transport Benign and sustainable additives Caveat: Moisture uptake, batch variation, and high-voltage compatibility
Sandwich architecture	Reinforcing or nonflammable core, self-shutdown	Set by core	Abuse tolerance without large polarization	Low-resistance bonding, in situ locking	High: Localized fire protection and self-shutdown Skins preserve transport while core handles heat Caveat: Extra interfaces increase resistance
Janus architecture	Face-specific chemistry for anode and cathode	Modest per face	Interface control with bulk transport kept	Correct orientation, graded porosity, in situ adhesion	High: Suppresses hot spots and dendrites while keeping conductivity Face-selective heat spreading and wetting Caveat: Correct orientation and adhesion required

## Data Availability

No new data were created or analyzed in this study. Data sharing is not applicable to this article.
